# Disinfection to control African swine fever virus: a UK perspective

**DOI:** 10.1099/jmm.0.001410

**Published:** 2021-09-21

**Authors:** Andrew D. Wales, Robert H. Davies

**Affiliations:** ^1^​ Department of Pathology and Infectious Diseases, School of Veterinary Medicine, Vet School Main Building, Daphne Jackson Road, University of Surrey, Guildford GU2 7AL, UK; ^2^​ Department of Bacteriology, Animal and Plant Health Agency, Woodham Lane, Addlestone, Surrey, KT15 3NB, UK

**Keywords:** African swine fever, disinfection

## Abstract

A review of African swine fever (ASF) was conducted, including manifestations of disease, its transmission and environmental persistence of ASF virus. Findings on infectious doses of contemporary highly-pathogenic strains isolated from outbreaks in Eastern Europe were included. Published data on disinfectant susceptibility of ASF virus were then compared with similar findings for selected other infectious agents, principally those used in the UK disinfectant approvals tests relating to relevant Disease Orders for the control of notifiable and zoonotic diseases of livestock. These are: swine vesicular disease virus, foot and mouth disease virus, Newcastle disease virus and *Salmonella enterica* serovar Enteritidis. The comparative data thus obtained, presented in a series of charts, facilitated estimates of efficacy against ASF virus for some UK approved disinfectants when applied at their respective General Orders concentrations. Substantial data gaps were encountered for several disinfectant agents or classes, including peracetic acid, quaternary ammonium compounds and products based on phenols and cresols.

## African swine fever virus: the role of the environment

### Disease and transmission

African swine fever (ASF) is a haemorrhagic disease affecting all ages of domestic and wild pigs, showing very high mortality during infection with virulent strains, and is caused by a double-stranded DNA virus. ASF virus (ASFV) was once regarded as an iridovirus but in recent years it has been reclassified, becoming currently the sole member of the family *Asfarviridae* [1].

ASFV replicates in mononuclear phagocytic cells and reticuloendothelial cells [1]. Clinical manifestations are varied, and range with strain virulence from sudden death (peracute form) through fever, skin reddening, cyanosis, anorexia, vomiting and diarrhoea culminating in death (acute form) to lower mortality variants (chronic form) showing depression, reduced appetite, mild and/or intermittent fever, skin lesions and arthritis [2]. Survivor pigs can shed virus for weeks to months depending on strain virulence [3, 4], although the capacity of longer-term carriers to transmit the disease is uncertain [5].

The disease is long-standing and endemic in southern and eastern Africa, where warthogs (*Phacochoerus africanus*) and bushpigs (*Potamochoerus porcus*) act as asymptomatic reservoirs of infection. In other parts of the world to which ASF has spread, wild boar and feral pigs are also an established reservoir for the virus, constituting a risk factor for introduction into non-biosecure pig units [6, 7]. The persistence of ASFV in wild populations is likely a consequence of several factors. These include: high lethality, relatively low contagiousness, and durability in carcasses plus the environment [8].

Infection occurs via oral or nasal exposure, and also via cutaneous wounds and tick vectors [4]. Airborne transmission can also occur over short distances [9, 10]. Tick vectors are of the genus *Ornithodoros*, and the endemic situation in Africa is associated with ticks of the *Ornithodoros moubata* complex living in association with warthogs [11]. *Ornithodoros erraticus* occurs in localised areas of some Mediterranean countries [12], but ticks of this genus are neither native nor recently reported in the UK [13, 14]. Mechanical transmission by other insect and tick vectors has been postulated [15] and viral DNA was commonly detected in stable flies and biting midges on infected premises [16]. An infection route that bypasses mucosal barriers via biting arthropods is plausible, given the low infectious dose via this route [5]. The documented persistence of viable ASFV for several weeks in frozen, cured and dried pig meat [3] illustrates the risk of importing the disease if pigs are allowed to consume contaminated products of this nature.

The infectious dose of ASFV appears to vary by strain [17, 18]. Blood is a potent source of virus for 3 to 4 weeks in non-lethal infections, and the infectious dose via skin wounds was found to be strikingly low (more than 10^4^-fold lower) compared with an oro-nasal dose of around 10^4^ 50 % haemadsorbing units (HAU_50_) for a moderately virulent isolate, as reported by McVicar [19]. More recent studies have shown that highly-pathogenic ASFV strains isolated from Eastern Europe reliably establish lethal infection following oral and/or nasal inoculation with much lower doses, of the order of 10^1^ HAU_50_, of young (two- to four month-old) domestic pigs [20] and weak or debilitated wild boar [21]. Moreover, the form of oral exposure appears to influence the infectious dose, as experimentally the dose needed to establish infection is several orders of magnitude higher in feed than in liquid [18]. This might reflect better preservation of virus viability in liquid, or possibly differing exposure of tonsils to the passing material.

The shedding of moderately-virulent virus in faeces and urine, in amounts sufficient to establish infection via the oral or nasal routes, has been observed experimentally to occur for around 1 week after the onset of fever [19]. Substantial peak titres of virus have been observed in samples from infected animals in various studies: 10^2^ to 10^5^ ml^−1^ in urine, 10^2^ to 10^6^ ml^−1^ in tonsil and nasal samples, and 10^2^ to 10^8^ ml^−1^ in rectal swabs [10, 19, 20, 22, 23]. Highest titres typically are seen when bloodstaining is present in such samples, and blood samples have the most continuous and consistent titres in acute disease, with reported maximal titres in the range 10^6^ to 10^9^ ml^−1^ [19, 20, 22].

### Environmental persistence of African swine fever virus

ASFV has a complex multi-layered structure, incorporating a lipid membrane between an inner core shell and an outer capsid. An outer lipid envelope surrounds the capsid [1]. The virus is robust in many environments, as illustrated by the following data on survival times.

Viable virus was recovered from blood for 70 to 192 days on the surface of boards, bricks or in soil at ambient temperatures. It was recovered after 105 days in putrefied blood and 540 days in blood at 4 °C (historical data, summarised in [3]). There was a reduction in HAU_50_ units over 3 weeks at 25 °C of up to two (but often less than one) log_10_ cycles in defibrinated blood from infected wild boar [24]. ASFV in spleen tissue from a naturally-infected animal showed a viability half-life of around 5.6 days at 4 °C and 0.7 days at 23 °C; equivalent values for virus in lung tissue were 6.3 and 0.4 days, respectively [25].

In faeces, ASFV was infectious by oral exposure after 11 days at room temperature in the dark [26]. Persistence in faeces in excess of 100 days is also claimed by certain secondary sources [3, 27, 28], although conditions of storage and subsequent inoculation/testing are not given. The virus in urine was infectious by oral exposure for at least 2 days after its removal from an infected carcase, but the efficiency of transmission declined thereafter [26]. ASFV was also viable after 45 days in urine buried in a glass flask [3]. Pig slurry spiked with ASFV and held at 4 or 22 °C showed an initial decline in HAU_50_ of around one log_10_ cycle after 5 h, but counts then remained similar when repeated 20 h later [29]. Over a longer period, and using ASFV adsorbed onto plastic carriers that were immersed in pig slurry, a decline of three log_10_ cycles was observed in 70 days at 17 °C; at 4 °C the same reduction occurred by 84 days, as reported by Haas *et al*. [27], citing unpublished findings by Eizenberger *et al.* Interestingly, contact with slurry appeared to slow down inactivation of plastic-adsorbed virus, compared to carriers that were also immersed in slurry but protected from direct contact with it.

The half-life of viable ASFV was found to be between four and 14 days in various feed matrices under simulated intercontinental transit time-temperature profiles [30–32]. In a related study, ASFV-spiked feed was found to have the virus titre reduced by around 1.3 log_10_ cycles after being subjected to a 30 day trans-Atlantic transport temperature simulation [33].

ASFV is generally quickly inactivated outside the pH range 4 to 11, but when in association with protein (serum), the virus is stable for hours below pH 4 and for days at pH 13.4 [34]. In a soil matrix, both pH and organic matter contribute to substantial variation in the duration of virus viability. At 25 °C, Carlson *et al.* [24] were unable to retrieve viable virus from acidic forest soils (pH up to 4.1) immediately after inoculation of around 5×10^6^ HAU_50_ in blood. By contrast, in soil with a neutral pH viable virus could be recovered for between one and two weeks after inoculation of a similar or smaller viral load. Intermediate stability was seen in mildly-acidic (pH 5.1) swamp mud. Low organic matter matrices yielded viable virus after inoculation for up to a week (beach sand) or several weeks (sterilised sea sand). By contrast, and possibly illustrating the protective effect of blood or other protein-rich tissue fluids, Mazur-Panasiuk and Woźniakowski [25] reported that when approximately 10^6^ HAU_50_ ASFV was introduced without blood to soil and leaf litter samples, infective virus was not recovered by 3 days after inoculation, across a wide range of storage temperatures.

Despite there being several assertions in the literature that sunlight is associated with inactivation of ASFV, quantitative data to support this seem to be lacking, or at least inaccessible. A Russian study reported no reduction in ASFV infectivity after 3 h of exposure to sunlight [35], although further details of the study are not readily available. It is very plausible that a tropical or sub-tropical intensity of sunlight (visible and ultraviolet) would reduce the time that exposed ASFV is viable, although it may be difficult to separate the effects of exposure to sunlight from desiccation under these conditions. In the 1990s in Sardinia, where free-ranging pigs were considered to be the principal ASFV reservoir, there was a strong seasonality to endemic ASF, with lowest incidence in the summer months [36]. This may have been influenced by reduced environmental persistence of the virus under the influence of more intense solar radiation, although this seems unlikely to be the sole factor.

### Persistence of infectious concentrations of virus in pig accommodation

Infection in pig units occurs in cycles, often with initial cases not diagnosed as ASF because the illness can appear non-specific or it presents as sudden death. Therefore, preventing spread and recycling of the virus within an affected unit is dependent on biosecurity and hygiene [4].

Based on published infectious doses, stored samples of ASFV-positive faeces from experimentally-infected animals were likely to be infectious via the oral/nasal route for around 5 days at 21 °C and over 8 days at 4 °C [23]. Likewise, for stored ASFV-positive urine, virus was present at probable infectious doses for at least 4.8 days at 21 °C and over 15 days at 4 °C. This data is broadly consistent with experimental studies, detailed below, on persistence of infectivity in uncleaned accommodation.

In the original report of ASF from 1921, Montgomery [26] found that pigs introduced into uncleaned accommodation occupied previously by affected pigs (ill to the point of death), themselves became infected if introduced at 3 days post-depopulation, but not at five and a half days. A recent study [37] also found that pigs became infected with virulent ASFV when moved into uncleaned accommodation previously occupied by experimentally-infected pigs, but only at 1 day post-depopulation and not at 3 days. In addition to strain variation, the virus load in the latter study may have been lower compared with the former, as pens were cleaned in the days before the final (depopulation) day, and departing pigs were euthanased at an earlier stage of clinical disease rather than allowed to die *in situ*.

It is worth noting that these studies used small numbers of healthy pigs on a few occasions. The data, discussed earlier, on virus concentrations, stability in blood, variation in infectious dose between strains, plus the efficiency of transmission via skin wounds and to weak or debilitated individuals, suggest that there may be occasions when ASFV can infect animals after substantially longer in the environment.

### Cleaning and disinfection

A recent review of cleaning and disinfection for ASFV has been published [38]. Its findings and recommendations are mostly not specific for ASFV, because of a lack of relevant ASFV disinfection data in the literature. However, one specific recommendation is for storage of slurry for 60 days or treatment with 2 % sodium hydroxide at 15 litres per cubic metre (0.03 % final concentration), as detailed in an Italian ASF outbreak plan [39].

Experimentally, sodium or calcium hydroxide added to slurry to a final concentration of 0.5 % reduced ASFV titres by at least three log_10_ cycles within 30 min at 4 and 22 °C [29]. More dilute sodium hydroxide (0.2 %) was similarly effective only at the warmer temperature. Rapid inactivation (more than four log_10_ cycles in 2.5 min) was seen with 1 % of either chemical, at 4 °C. The same study concluded that heating slurry to 60 °C caused a more than four log_10_ cycle titre reduction of ASFV in 2 to 4 min. Heat can also be used to help decontaminate pig transport lorries [40] and combination of heat and biocidal treatment can often be synergistic [41].

The World Organisation for Animal Health (OIE) recommendations for disinfectant agents against ASFV [2] are:

- Sodium hydroxide, 0.8 % for 30 min
- Hypochlorite, between 0.03 and 0.5% available chlorine for 30 min
- Ortho-phenylphenol, 3 % for 30 min
- Formalin, 0.3 % for 30 min
- Iodine compounds (unspecified)

The United States Department of Agriculture (USDA) also maintains a list of disinfectants approved for use against ASFV in farm settings [42]. It currently comprises Environmental Protection Agency (EPA) approved commercial products based on: chlorine, hydrogen peroxide, and potassium peroxymonosulphate (Virkon-S). Only if a commercial, approved preparation is not available is it then permitted to use sodium hypochlorite (0.3%) or citric acid (3%). For both, exposure times after cleaning are 15 or 30 min on non-porous and porous surfaces, respectively. Thymol, a monoterpene phenol believed to act on membranes, is also permitted, at a concentration of 0.05%, in the absence of an EPA approved product. Its use in this context is restricted to disinfection of hard nonporous surfaces of aircraft and associated loading equipment.

## Published data on disinfectant action on African swine fever virus and other selected biological targets

### Context and method

Published data on disinfection of pathogens was examined in the context of the UK Department of Environment, Food and Rural Affairs (Defra) approval tests. The Defra disinfectant approvals scheme ensures the efficacy of disinfectants used to control notifiable and zoonotic disease in GB. Disease Orders, allowed for in the Animal Health Act 1981, include requirements for disinfection. Approval of disinfectants for the various Orders is underpinned by the Diseases of Animals (Approved Disinfectants) (England) Order 2007. Currently, five Orders categories are specified: Swine Vesicular disease (SVD) virus Orders, Foot and Mouth disease (FMD) virus Orders, Tuberculosis Orders, General Orders (G.O.), and Diseases of Poultry (DoP) Order plus Avian Influenza and Influenza of Avian Origin in Mammals (AI and IAOM) Order. G.O. cover disinfection to control all animal diseases notifiable in GB which are not covered by the other four disease-specific orders.

Suspension tests at low temperature (4 °C) are used to test disinfectant products and to establish in-use concentrations of them for each of the Orders categories. The reference organism for the G.O. test is *Salmonella enterica* ser. Enteritidis (‘*S.* Enteritidis / SE’). Conditions and test targets for the various approvals [43] are shown in Table 1. It can be seen that, compared with the virus tests, the G.O. test uses a lower test mix disinfectant concentration and a higher (five log_10_ cycles titre reduction) pass threshold. The SVD and FMD Orders tests also have less organic soil. These features probably contribute to the pronounced tendency for G.O. concentrations to match or exceed the concentrations for other disease orders. In the current Defra list the G.O. concentration is equal to or higher than that for FMD, SVD and DoP/AI and IAOM Orders in 83, 59 and 82 % of cases, respectively.

**Table 1.** Details of Defra disinfectant approval tests

Orders	Test organism	Other details
Diseases of Poultry plus Avian Influenza and Influenza of Avian Origin in Mammals	Newcastle disease virus strain Herts 33	WHO hard water diluent, 2.5 % baker’s yeast soil, 4 °C, 30 min contact time halted by neutralisation, performance standard is ≥4 log_10_ unit reduction in egg infective doses
Swine vesicular disease	Swine vesicular disease virus UK (British field strain) G 27/72	WHO hard water diluent, 4 °C, 30 min contact time halted by neutralisation, performance standard is ≥4 log_10_ reduction.
Foot-and-mouth disease	Foot- and-mouth disease virus O1 BFS (British field strain) 1860/UK/67	As SVDV test, plus 1 % foetal bovine serum soil
Tuberculosis disease	* Mycobacterium fortuitum *	Follows British Standard BS 6734 : 2004, 4 °C, 2.5 % yeast soil, 60 min contact time halted by neutralisation, in-use dilution, *mixed 1 : 1 with inoculum*. Performance standard is ≥4 log_10_ unit reduction
General	* Salmonella* Enteritidis S9574/07	Based upon a former British Standard (BS 6734 : 1986); 4 °C, 2.5 % yeast soil, 30 min contact time halted by neutralisation, in-use dilution, *mixed 1 : 1 with inoculum*. Performance standard is ≥5 log_10_ unit reduction

The primary aim of the present data review was to obtain published, quantitative data on the effect of well-defined disinfection agents upon ASFV and compare it with similar data for biological targets used in the Defra approval scheme. These targets are viruses of FMD (FMDV), SVD (SVDV) and Newcastle disease (NDV), plus *S.* Enteritidis. By this method, the likely efficacy of Defra approved disinfectants against ASFV may be estimated. Ancillary data relating to avian influenza virus (AIV), parvoviruses, a pig coronavirus (transmissible gastroenteritis virus, TGEV) and human respiratory coronaviruses of the SARS-CoV/SARS-CoV2 group were sought also. Characteristics of all viruses are summarised in Table 2.

**Table 2.** Selected characteristics of viruses used in the reported disinfectant tests

Virus*	Characteristics	References
ASFV	Family Asfarviridae, genus *Asfivirus*; dsDNA, 175–215 nm, enveloped, complex multilayer capsid. See text for other detail.	[5]
FMDV	Family Picornaviridae, genus *Aphthovirus*; ssRNA; small (30 nm), non-enveloped. Increasingly labile outside pH 7.0–8.5. Resists detergents and organic solvents. A proportion of viable virus remains after drying, with enhanced survival in organic matter.	[80, 82]
SVDV	Family Picornaviridae, genus *Enterovirus*; ssRNA; small (30 nm), non-enveloped. Stable in the pH range 2.5–12.0. Can survive for long periods in the environment. Low susceptibility to many disinfectants.	[47, 77, 80, 83, 84]
NDV	Family Paramyxoviridae, genus *Avulavirus*; ssRNA; ≥150 nm, enveloped. Inactivated by acid pH ≤2, survives for long periods at ambient temperature. Sensitive to detergents, lipid solvents, formaldehyde and oxidizing agents.	[85–87]
AIV	Family Orthomyxoviridae, genus *Influenzavirus A*; ssRNA; 80–100 nm, enveloped. Viable for days in environment, susceptible to a wide variety of disinfectants including surfactants, and inactivation at pH values below 3 and above 10.	[88, 89]
Parvo-viruses (non-human)	Family Parvoviridae; ssDNA; small (18–26 nm), non-enveloped. Resistance to low pH is often marked, but varies between types. Survive up to 1 year in organic material. Moderate susceptibility to peracetic acid, low susceptibility to many other disinfectants.	[81, 90, 91]
TGEV	Family Coronaviridae, genus *Alphacoronavirus*; ssRNA; approx. 130 nm, enveloped. Stable between pH 4 to 8, with strain variation. Environmental (in-feed, water and slurry) survival for days to weeks. Disinfectant susceptibility likely broad but data patchy.	[27, 92–94]
Sars-CoV	Family Coronaviridae, genus *Betacoronavirus*; ssRNA; approx. 130 nm, enveloped. Stable between pH 5 to 9. Survival in body fluid and faeces for several days at room temperature. Broadly disinfectant-susceptible, including surfactants.	[93–95]

*ASFV, African swine fever virus; FMDV, foot and mouth disease virus; SVDV, swine vesicular disease virus; NDV, Newcastle disease virus; AIV, avian influenza virus; TGEV, transmissible gastroenteritis virus; Sars-CoV, SARS-CoV-1 virus.

Suspension test data was considered separately from surface test data, as factors influencing disinfectant activity differ substantially between the two types of test [44]. Defra approval does not currently use surface tests. Nonetheless, data from such tests showing that an agent was similarly or more effective against ASFV than against a Defra test pathogen (SE, FMDV, SVDV, NDV) was considered valuable, as it was taken to suggest that similar relative efficacy would be observed in field application.

Concentrations of disinfectant agents in suspension tests were calculated as that present in the final mix with virus or *Salmonella *, and in surface tests as that applied to the test surface. Virus titres were given in the cited studies, variously, as: 50 % tissue culture infective dose (TCID_50_), 50 % egg infective dose (EID_50_), 50 % egg lethal dose (ELD_50_), or plaque-forming units (pfu), according to the methods of propagation and detection of virus titre employed.

Organic soil loads differed substantially between studies, both in quantity and composition. To allow some comparison between studies, soil loads were classified into five categories (zero, light, intermediate, heavy, very heavy), based principally on expected protein concentration, either in the final test mix for suspension tests or in the undried surface inoculum for surface tests. Further detail is in Table 3. Classification boundaries were selected with reference to soil loads used in disinfectant test standards published by organisations either with an international reach (European Committee for Standardization/CEN, ASTM International, Organisation for Economic Cooperation and Development/OECD) or a veterinary focus (Deutsche Veterinärmedizinische Gesellschaft/DVG). Among these standards, soil loads for veterinary applications typically are higher than for other uses. The G.O. test soil level (2.5 % sterilised yeast) is classified as ‘high’ in this scheme.

**Table 3.** Classifications used for amount and type of added organic soil in disinfection tests

Classification	Key composition	Actual mixes used
Zero	No added material	
Light	Protein/peptides 0.015–0.1 %	Peptone 0.15 % Bovine serum albumin 0.03 % Allantoic fluid Bovine serum 1 % Foetal calf serum 1 % or 2.5 %
Intermediate	Protein/peptides >0.1 to 1 %	Bovine serum albumin 0.3 % Bovine serum albumin 0.1 %, plus yeast extract 0.1 % Foetal calf serum 5 % Bovine serum, 0.25 %, plus mucin 0.08 %, plus yeast extract 0.35 %
Heavy	Protein/peptides >1 %	Bovine serum 20 % Foetal bovine albumin 1%, plus yeast extract 1.5%
Very heavy	Blood or faeces 20–75 %	Pig blood 75 % Pig faeces 20 % or 75%

Data, discussed earlier, imply that the concentration of ASFV shed into the environment by some infected pigs (via secretions, urine, faeces and blood) can readily deliver an infectious dose to a susceptible individual upon exposure to even a small amount of such material. Therefore, disinfection to control and eliminate ASF should aim to achieve a reduction of several log_10_ cycles in virus titre. In this context, the threshold criterion for UK Defra virucidal tests, of a four log_10_ cycle reduction in virus titre (Table 1), seems an appropriate minimum.

For any given disinfectant, evidence that ASFV is at least as susceptible as *S.* Enteritidis is valuable information. It implies that a G.O. concentration of that disinfectant would satisfy a G.O. -type suspension test of ASFV, i.e. a reduction in virus titre of at least five log_10_ cycles, with a one-to-one disinfectant and inoculum mix plus heavy organic soil in the final cold (4 °C) test mix. Such a relative susceptibility between *S.* Enteritidis and ASFV increases confidence that there would be sufficient virucidal activity under G.O. application to livestock accommodation. This is a challenging disinfection environment, where variations in surface materials, ambient temperature, organic soil, exposure time and diluting water will be encountered.

**Fig. 1.**
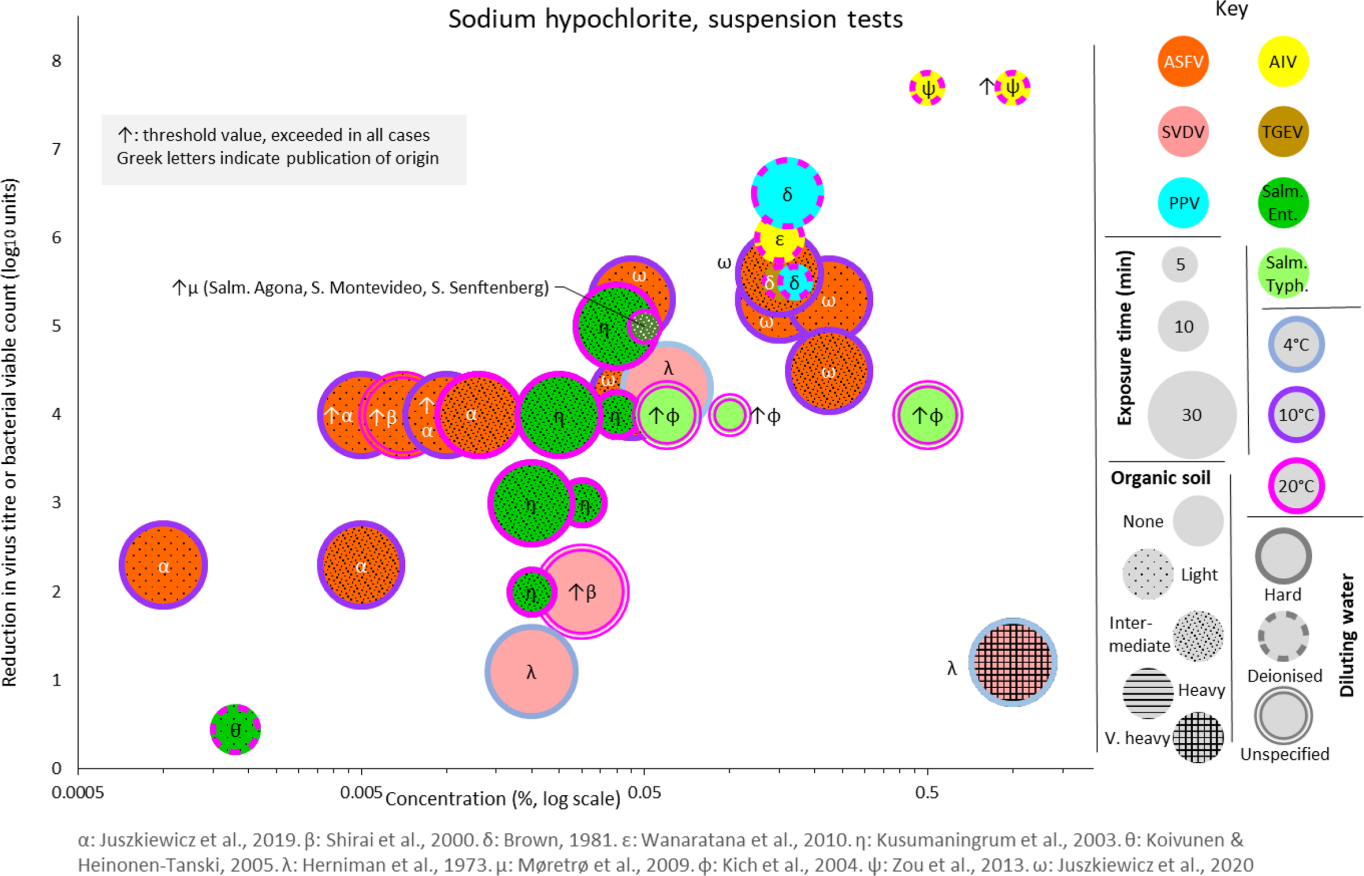
Specimen bubble chart used for data analysis

Where published data was found in an appropriate form to allow comparisons of the above factors, bubble charts were produced for suspension and/or surface tests in order to facilitate comparative assessments involving the numerous variables encountered in the published studies. As these are not straightforward to interpret, because of the several encoded variables, only one is included here, as a specimen example of this visual tool for analysis (Fig. 1). However, all such charts, plus a master key, are included in the Supplementary material (available in the online version of this article).

### Disinfectant agent data

#### Oxidising agents

##### Sodium hypochlorite

Sodium hypochlorite (NaOCl) is the agent with the largest volume of data that is sufficiently well-defined, particularly in respect of concentration used, for comparisons between its effects on targets (including ASFV) to be made. The principal active chemical species formed by sodium hypochlorite in aqueous solution are: hypochlorite anion, hypochlorous acid and chlorine [45]. All of these are considered to represent ‘active’ or ‘available’ chlorine, although hypochlorous acid is the most powerful oxidising agent of the three, and antimicrobial activity is higher in the neutral to low pH range, dominated by hypochlorous acid and/or elemental chlorine. All concentrations from cited material, if not already expressed as percent w/v, were converted to these units, according to published conversion scales [46]. As percent NaOCl and percent available chlorine are nearly equivalent (the molecular mass of NaOCl being similar to that of Cl_2_), and as in some papers it is not clear which of these percentages was being used, the two are treated as equivalent for the purpose of comparisons.

For suspension tests (Figs 1 and S2), there is suitable comparative data for SVDV, AIV, porcine parvovirus (PPV), TGEV, and *Salmonella enterica* serovars Enteritidis and Typhimurium. SVDV appears to be less- or similarly-susceptible than ASFV under similar conditions of exposure time and water quality, even when organic soil is added to ASFV [47–50]. This suggests that chlorine disinfectants approved for SVD Orders are likely to be efficacious against ASFV, although one caveat is that all the SVDV data arise from low-temperature (4 °C) tests whereas ASFV data are from 10 to 20 °C tests. Findings for the other veterinary viruses [51–53] are difficult to compare with ASFV, as the concentration of available chlorine used was generally substantially higher for them in the data sources available.

*S.* Enteritidis in suspension appears to be broadly as susceptible as ASFV to inactivation by hypochlorite solutions under similar conditions of exposure time (30 min), temperature (10–25 °C), and hard water [54]. These are warmer test conditions than for G.O. approval (4 °C), but they suggest that G.O. application of chlorine-based disinfectants in the absence of heavy organic soiling should have substantial ASFV-inactivating effect. However, the data for ASFV and *Salmonella* were derived from different studies and when comparing ASFV with *S.* Enteritidis there are few instances where all of the temperature, soil and hypochlorite concentration match closely. Therefore this can only be a cautious inference. Data for shorter exposure times (i.e. 5 to 10 min) are not available for ASFV [54, 55]. Data for *Salmonella* ser. Typhimurium [56] suggests a higher susceptibility than for *S.* Enteritidis.

Among surface tests (Fig. S3), the largest amount of comparative data is available for FMDV on stainless steel, wood and plastic surfaces [57–59]. There is an additional benefit that data for ASFV and FMDV are derived from the same studies, decreasing the possible impact of method upon observed differences. On these surfaces and with light organic soil and hard water diluent, FMDV appears consistently to be less susceptible than ASFV to chlorine-based disinfection at room temperature. For each virus, both stainless steel and polystyrene substrates yielded similar results, whereas wooden surfaces require more time and/or concentration for an equivalent effect. FMDV also appears to be less susceptible than ASFV on heavily-contaminated fresh and acrylic-sealed concrete surfaces. Fresh concrete, which would likely have a higher surface pH than sealed or aged concrete [60], appears to enhance the virucidal effect for ASFV. Limited inactivation was observed for both viruses in the presence of heavy organic material contamination on stainless steel, consistent with the known susceptibility of hypochlorite to interference by organic matter. Thus, FMD Orders approved chlorine-based disinfectant application appears likely to have similar or greater effect on surface-located ASFV compared with FMDV. Consequently, where a chlorine disinfectant is approved for G.O. at a concentration at least equal to FMD Orders, the G.O. rate is likely to be effective, in a sufficiently clean environment.

Data for AIV is more fragmentary, and appears somewhat inconsistent between sources. Some results suggest similar susceptibility compared with ASFV in the 0.04–0.05 % concentration range [61], whilst lesser susceptibility of AIV is seen at higher hypochlorite concentrations, albeit from a different study [62]. This may reflect methodological or strain differences. NDV appears to have similar or lower susceptibility compared with ASFV, although the data for NDV is very limited [62]. Surface test results for *Salmonella* spp. [63, 64] include one for serovar Enteritidis on stainless steel [65], but this last study does not allow for confident comparison with ASFV. There are differences in organic soil, uncertainty about the diluting water and a ‘carrier’-type methodology was used, with coupons being immersed in *Salmonella* suspension then immediately transferred into excess disinfectant. By contrast, for ASFV on steel and plastic a ‘surface test’ methodology was used, where the target organism was dried onto the coupon then overlaid with a defined small volume of disinfectant [57, 59].

The USDA Animal and Plant Health Inspection Service (APHIS) has approved sodium hypochlorite to be used against ASFV in farm settings, at 0.3 %, as discussed earlier. In the currently-collated suspension test data, such a concentration is associated with a reduction in virus titre exceeding four log_10_ cycles in the presence of moderate soiling. Surface test data suggests similar efficacy even on wood [58], although low-temperature (4 and 10 °C) performance data is lacking.

##### Peroxygen compounds (excluding hydrogen peroxide)

Among suspension test results, ASFV inactivation data (Fig. S4) arose from conditions (temperature, disinfection agent, concentration, soiling) that in most cases were not closely similar to those reported for other key viruses (SVDV, FMDV AIV) [47, 49, 50, 66]. However, in one instance (heavy organic soil, room temperature, 10 min exposure), FMDV appeared to be more susceptible than ASFV to potassium peroxymonosulphate plus surfactant (likely ‘Virkon-S’) [59]. Comparative data from two studies [55, 64] suggest a substantial difference between the two *Salmonella* serovars Enteritidis and Typhimurium in susceptibility to peracetic acid, at least with the two Typhimurium and one Enteritidis strains tested. This is a pattern also evident in the data for hypochlorite. It implies that data for serovar Typhimurium cannot be taken as a reliable guide to the effect of application of a disinfectant at a G.O. concentration, as this is derived from testing with a serovar Enteritidis reference strain.

AIV appears more susceptible than ASFV to potassium peroxymonosulphate in both suspension and surface tests (Figs S4 and S5) [49]. Surface tests with *S.* Enteritidis [65] were not similar enough in respect of agent or concentrations to draw any confident conclusions on effects of G.O. approved peroxygen disinfection of ASFV. However, a surface disinfection study including peracetic acid (PAA) and four parvoviruses (bovine, porcine, murine and canine; Fig. S5) provides some data for ‘resistant’ viruses (Table 2) compared with *S.* Enteritidis [67]. This suggests that an application of PAA that achieves multiple log_10_ cycle reductions in viable *S.* Enteritidis will have a similar effect on porcine, murine and bovine parvovirus (canine parvovirus appears more resistant). Therefore, if ASFV is no more resistant to PAA than these three parvoviruses, then G.O. concentrations should be efficacious.

##### Iodides

Suitable AFSV data was only found in results of suspension tests (Fig. S6) [48, 56, 68]. The principal relationship evident is greater susceptibility of ASFV than SVDV to a solution of an iodoglycine complex (potassium tetraglycine triiodide), with both results coming from the same study. This suggests that SVD Orders approved iodine-based disinfectant would have similar or greater effect on ASFV than on SVDV (although data is sparse). Given the difference in iodine formulations, it is difficult to conclude much about the relative susceptibilities of ASFV and *Salmonella *. In consequence, suitable data to predict the effect of G.O. approved iodine disinfection on ASFV has not been found, except where the disinfectant also has SVD Orders approval.

##### Hydrogen peroxide

Heckert *et al.* [69] examined hydrogen peroxide vapour (1200 ppm, 30–40 °C) in contact with target viruses, including ASFV, suspended in small volumes (100 µl) of water with organic soil. Results (Fig. S7) indicate that ASFV and AIV are similarly-susceptible, and that both are less susceptible to inactivation under these conditions than NDV or SVDV. Surface disinfection tests (Fig. S8) using a commercial hydrogen peroxide product (‘Intervention’) at its label concentration showed modest performance (less than a two log_10_ cycle reduction on average) after 10 min’s exposure on steel. There was still-poorer performance on concrete surfaces, which had been artificially aged (carbonated) to reduce surface alkalinity [70]. The data found on NDV susceptibility [71] was derived from concentration and time conditions that are too different from those relating to ASFV to make a confident comparison on susceptibility.

##### Virkon S

‘Virkon S’ is the only commercial disinfectant formulation for which there are several suitable studies comparing efficacy and among which ASFV is included as a target. Ingredients in Virkon S include: potassium peroxymonosulphate, sodium chloride, organic acids, inorganic buffers and anionic surfactant. It is principally an oxidising disinfectant. When made up, the mix of biocidal ingredients is acidic, and is augmented by hypochlorous acid created by the internal chemistry of the product [72].

Appropriate data was found for surface tests only (Fig. S9). ASFV appears to be less susceptible than FMDV to inactivation on steel surfaces under conditions of intermediate soil and a normal (1 %) Virkon S concentration [60], and also at 2 % concentration with very heavy soil [59]. Therefore, both suspension and surface data for peroxymonosulphate-based preparations suggest that FMDV-approved disinfectants at the FMD-approved dilution may not perform as well against ASFV. These results may reflect the known acid-sensitivity of the latter virus [60, 70]. Two data points from the same study (labelled ‘↑SS α’ in Fig. S9) illustrate this; the FMDV test showed no detectable virus in eight repetitions whereas the ASFV test showed residual virus in one of four repetitions. There appears to be a similarly lower susceptibility of ASFV to Virkon S compared with FMDV, when tested with intermediate soil on carbonated concrete surfaces. Comparing surfaces rather than targets, a substantially lower ASFV virucidal effect of Virkon S (at the usual working concentration of 1 %) was seen on carbonated concrete than on stainless steel [70].

The single test involving SVDV (not from a study where ASFV was also tested) suggests this virus may, similarly to FMDV, also be more susceptible than ASFV to surface-applied Virkon S [73], although this must be regarded as speculative, given the sparsity of data. Results for NDV and AIV also indicate that ASFV may be less sensitive than these viruses to Virkon S, at least on steel surfaces [62, 71]. Therefore, on the basis of the present data, it cannot reasonably be assumed that ASFV inactivation by Virkon S will be similar to or greater than inactivation of FMDV, SVDV, AIV or NDV under similar surface-applied conditions.

The two *Salmonella* serovars in the data (Senftenberg and Agona) appear to be similar to or more resistant than ASFV, in terms of susceptibility on steel, although they were tested with hard water diluent which may have reduced their susceptibility [63]. Closely-similar tests (temperature, diluent, soil, and concentration) were not found for comparing ASFV and *Salmonella* inactivation by Virkon S, and none were found involving the G.O. test serovar (*S.* Enteritidis).

### Acids

#### Citric acid

Only surface test data was found for citric acid (Fig. S10). Interestingly, given that FMDV is known to be highly sensitive to citric acid, the present data suggest that both it and ASFV appear to have a similar sensitivity to this agent, on both steel and wood surfaces and with light or heavy organic soil [57–59]. There was much less inactivation of ASFV on concrete compared with steel, and this was even after the concrete had been artificially aged through carbonation [70]. This possibly reflects the high surface pH of even carbonated concrete. Data for AIV suggests that it is similarly or more susceptible to citric acid than ASFV, although a close comparison on the same (steel or plastic) substrate with equivalent soiling is not available [61, 66]. It is difficult to conclude much about the comparative susceptibility of ASFV and NDV with the present data [71].

Citric acid is not approved under the EU Biocidal Products Regulation (BPR) for veterinary hygiene (Product Type 03), so there are no FMD Orders or G.O. approved citric acid concentrations to compare with ASFV susceptibility. By contrast, the United States APHIS has approved 3 % citric acid to be used against ASFV in farm settings under certain circumstances, as discussed under ‘Cleaning and Disinfection’. Comparison with the data in Fig. S10 suggests that such application is likely to be efficacious in achieving multiple log-unit reductions in ASFV titres on steel surfaces, although the high-pH surface of concrete may pose a stiffer challenge.

#### Acetic acid

Acetic acid was tested in suspension using light and moderate soil, at 10 °C for 30 min by Juszkiewicz *et al.* [50]. ASFV was substantially inactivated (>4 log_10_ reduction of virus titre) at 3 and 2% acid concentration with moderate and light soil, respectively.

### Sodium hydroxide (caustic soda)

Suspension test data was found for ASFV, SVDV and Porcine Parvovirus [47, 50, 51]. However, each virus was tested in a different study from the other two, and conditions of soiling, water quality and temperature were dissimilar (Fig. S11). Concentrations of 2 and 3% sodium hydroxide were associated consistently with greater than four log_10_ cycle reductions in ASFV titres after 30 min’s exposure at 10 °C with light or moderate soiling [50].

### Quaternary ammonium compounds

The variety of different quaternary ammonium compounds (QAC) tested, alone or in combination with other chemicals, reduces the opportunity for direct comparisons between different studies. In suspension (Fig. S12), didecyl dimethyl ammonium chloride (DDAC) was tested against ASFV (enveloped) and SVDV (non-enveloped) under similar conditions [48, 74]. ASFV appears to be substantially more sensitive to this compound, with SVDV showing much less inactivation, even to a tenfold higher working concentration than one used with ASFV. Thus, SVD Orders approved application of QAC-based disinfectant may be effective against ASFV also. The data also suggest that ASFV shows greater sensitivity to DDAC than does *Salmonella* ser. Typhimurium to another QAC (alkyl dimethyl ammonium chloride, ADAC) [56]. Data for effects of benzalkonium chloride on ASFV and *Salmonella* ser. Typhimurium suggest the latter is the more susceptible target [50, 64].

There is more data for surface tests (Fig. S13), which indicate that ASFV and FMDV were similarly-sensitive to a commercial QAC-plus-surfactant preparation, on steel, plastic and concrete surfaces, even with heavy soiling [59]. There may be differing mechanisms at work with compound disinfectants on these two very differently-constituted viruses (for example: membrane versus pH effects), so the exact formulation of QAC-based disinfectants might substantially alter the relative susceptibilities of FMDV and ASFV. Other comparisons between viruses [48, 52, 62], and with *Salmonella* spp. [64, 65], are hampered by undefined or differing constituents of the QAC preparations, although AIV and NDV appear similarly sensitive to ADAC.

### Aldehydes

#### Formaldehyde

The only study that reported formaldehyde as a sole test agent was unable accurately to gauge its virucidal effect because of toxic effects on the Vero cell detection system [50]. However, formaldehyde is considered to be a highly effective, broad-spectrum (including bacterial spores) biocide that is not readily inactivated by organic matter, although its routine use is limited by toxicity concerns in relation to operators [75, 76].

#### Glutaraldehyde

In suspension tests (Fig. S14), concentrations of glutaraldehyde between 0.1 and 1.0 % for 30 min at 10 °C with hard water plus light or moderate soil were associated with four to five log_10_ cycle reductions in ASFV titres [50]. SVDV appeared to be markedly less susceptible than ASFV to glutaraldehyde, both at room temperature and under cold (4 °C) conditions [47, 77]. Furthermore, SVDV data was obtained with disinfectant solutions that were verified as alkaline, under which condition glutaraldehyde has greatest biocidal activity [78]. Virus data from two additional studies showed AIV to be more susceptible than ASFV or SVDV [52, 66].

Under warmer (but otherwise similar) conditions to SVDV tests, *Salmonella* ser. Typhimurium (one reference strain, one field strain) appeared to be more susceptible than ASFV, showing equivalent or greater reductions in viable counts at lower disinfectant concentrations [64]. However, as discussed with the hypochlorite and peroxygen data, this on its own may not be a reliable guide to ASFV versus *S.* Enteritidis susceptibility, and therefore to the effect on ASFV of a G.O. concentration. In this circumstance, an SVD Orders concentration for a glutaraldehyde-based disinfectant would be very useful additional information to gauge the adequacy of application at a G.O. rate for destruction of ASFV.

### Phenolic compounds

In suspension, 1 % phenol appeared efficacious against ASFV in the presence of light or intermediate soil (Table 3), effecting reductions in virus titre of more than four log_10_ cycles. However, there was a marked threshold pattern, with 0.5 % phenol showing minimal virucidal effect under the same conditions [50]. A surface disinfection test using ASFV, an intermediate soil load (ASTM standard mix of bovine albumin, mucin and yeast extract, equivalent to 5 % bovine serum) and a commercial blend of phenolic compounds (‘Tek-Trol’) at the label dilution of 0.39 % (1 : 256) was reported by Gabbert and Neilan [70]. With a 10 min exposure at room temperature on stainless steel, a moderate reduction (around 2.7 log_10_ TCID_50_) in virus titre was observed. The reduction was attenuated to around 1.8 log TCID_50_ on a carbonated, unsealed concrete surface.

### Mixed-class disinfectants

Surface tests of two commercial mixes of QAC plus glutaraldehyde were reported by Gabbert and Neilan [70], for 10 min at room temperature with a moderate (ASTM standard albumin/mucin/yeast extract) soil load. One disinfectant (‘Virocid’) additionally incorporates isopropanol and pine oil, and at the label concentration of 1 : 256 (0.39 %) it effected a greater than four log_10_ cycle reduction of ASFV titre on both stainless steel and carbonated concrete. The other (‘Synergize’) was a simpler two-component mix and, when used at its label concentration (also 0.39 %), it appeared to be substantially less efficacious. Average titre reductions of 2.9 and 2.1 log_10_ cycles were observed on steel and concrete respectively, around two log_10_ cycles less than that observed with Virocid for each surface material.

### Disinfectant data: summary of findings

In suspension mixed with sodium hypochlorite, ASFV appears to be as susceptible as *S.* Enteritidis and more susceptible than SVDV. On surfaces, ASFV appears more susceptible to sodium hypochlorite than does FMDV (steel, wood, plastic and concrete). This suggests that General, FMD and SVD Orders approved applications of chlorine-based disinfectants will inactivate ASFV similarly to SVDV, FMDV and *S.* Enteritidis. Data from the other commonly-used halide disinfectant (iodine) indicates that ASFV is more susceptible to this agent than SVDV when tested in suspension.

A single study using vaporised, warm hydrogen peroxide indicated that ASFV and AIV were similarly susceptible to this agent, but less readily inactivated than either SVDV or NDV. Dissimilar data hamper comparisons for another peroxygen disinfectant (potassium peroxymonosulphate), but it seems likely that ASFV is less susceptible to it than FMDV or AIV. Correlating with this, a compound peroxygen-containing disinfectant commonly used in the pig industry (Virkon S) applied to steel or concrete inactivated ASFV less readily than it did FMDV, NDV or AIV. Surface disinfection data suggest that *Salmonella* (serovars Agona and Senftenberg) and ASFV are all similarly susceptible to Virkon S.

When tested against citric acid, ASFV and FMDV show a similar susceptibility on either steel or wood surfaces, but no comparative data for *Salmonella* spp. were found. The attenuating effect of concrete on the anti-ASFV action of this acidic disinfectant is clearly evident. Sodium hydroxide, being a high-pH disinfectant, may be less influenced by concrete surfaces, but data suitable to confirm this was not found.

A QAC (DDAC) appeared to be more active against ASFV in suspension when compared with SVDV. Against a QAC plus surfactant mix, ASFV and FMDV were similarly susceptible on steel, plastic and concrete. However, the exact chemical makeup of QAC agents and the composition of QAC products appear to influence virucidal activity substantially. Therefore an efficacious concentration for one QAC product seems unlikely to provide a reliable guide to a suitable concentration for a non-identical product.

For glutaraldehyde, the suspension test data of Juszkiewicz *et al.* [50] indicate that this may be an effective agent against ASFV, and may also be relatively resistant to attenuation by organic soil. Glutaraldehyde plus QAC is a commonly used disinfectant mix in the pig industry, and the evidence from both glutaraldehyde and QAC suspension tests is that SVDV is substantially less susceptible than ASFV to both agent types. Therefore, it is reasonable to speculate that SVD Orders-approved glutaraldehyde-plus-QAC disinfectants would likely be efficacious against ASFV at G.O. concentration, provided this is equivalent to or higher than their SVD Orders concentration. This extrapolation does not account for possible differences between the viruses in susceptibility to synergistic effects of the two biocide classes. Surface tests reported by Gabbert and Neilan [70] showed that for two examples of compound glutaraldehyde/QAC disinfectants, the exact composition (including additional biocide classes in one product) substantially influences efficacy against ASFV on surfaces at standard USA label (i.e. non-Defra) concentrations.

### Implications for Defra approved disinfectants

It is possible to compare General, FMD and SVD Orders concentrations of current Defra approved disinfectants [79] with the present data, and with those approved products for which information on their principal constituents is readily available. This allows the identification of some commercial disinfectants that are likely to perform effectively against ASFV at G.O. concentrations. This is presented in Table 4. It should be noted that most Defra approved disinfectants are not present in the table and this is typically because there isn’t readily-available data (for comparative effects, or on disinfectant composition) to make a prediction, rather than there being evidence that they are not potentially effective against ASFV. This includes the common biocide chemistries of peracetic acid, phenols, and cresols. In Table 4, it can also be seen where the data implies caution for some products, given the aforementioned need for reductions in virus titre of several orders of magnitude to be confident of preventing transmission. Such evaluations should be considered in the context of general strengths and weaknesses of disinfectant classes in the field, particularly the known susceptibility of halide disinfectants to inactivation by organic matter.

**Table 4.** Selected* Defra approved disinfectants with sufficient Disease Orders approvals to estimate efficacy against African swine fever virus

Disinfectant	G.O. dilution rate†	Chemistry	Comment
Quinticare +	2	Quaternary ammonium	**Caution:** weak evidence for likely efficacy, i.e. different QACs in the data comparing ASFV and SE effect, and a different QAC again in this formulation. No SVD or FMD Orders approvals/concentrations to provide support.
Agrichlor (Evans) Agrichlor (Hydrachem) Anigene NaDCC BioKlor BIOSPOT Virochlor 500	360	Chlorine	Fair evidence for likely efficacy. Reference data: SE and SVDV in suspension, and FMDV in surface tests. Compared with GO, FMD Orders use more dilute (1/424), SVD Orders use less dilute (1/317).
Credence 1000 Septrivet 17	299	Chlorine	Fair evidence for likely efficacy. Reference data: SE and SVDV in suspension, and FMDV in surface tests. General, FMD and SVD Orders all use the same dilution.
FAM 30 Farmsan Total Farm Disinfectant Virudine Plus	49	Iodophor	Higher susceptibility of ASFV than SVDV to potassium tetra-glycine triiodide in suspension, and G.O. 2× the concentration of SVD Orders (1/100). Therefore, fair evidence that G.O. concentrations likely to be efficacious against ASFV.
Bio-VX Medicide +	100	Peroxygen	ASFV moderately less susceptible to potassium peroxymonosulphate than FMDV, but G.O. 12× more concentrated than FMD Orders (1/1200). Therefore G.O. application likely to be effective against ASFV.
‘Virkon Professional’ ‘Virkon S’	100	Peroxygen plus chlorine	G.O. 13× and 2.8× more concentrated than FMD and Poultry (NDV) Orders, respectively, but FMDV highly susceptible and NDV more susceptible than ASFV. G.O. surface application may sufficiently inactivate ASFV, based on ≤10 min exposure and including *Salmonella* data (not Enteritidis), but better comparative data would improve confidence.
‘Virkon Professional’ tablets ‘Virkon S’ Tablets	100	Peroxygen plus chlorine	G.O. 10× and 2.5× more concentrated than FMD and Poultry (NDV) Orders, respectively. Additional comments as for other Virkon products.
Virocid	33	Glutaraldehyde, QAC, alcohol and pine oil	G.O. rate (c. 3 %) is more than 7.5× as concentrated as the rate shown to achieve >4 log_10_ reduction against ASFV on steel and concrete with moderate soil‡. Label rates 0.25–1.5 %.
Omnicide Omnicide FG Omnicide FGII	50	Glutaraldehyde plus QAC	Biocidal effect on ASFV likely to exceed that for SVDV at SVD Orders rate (100), given relative susceptibilities of ASFV and SVDV to both biocide classes in suspension. G.O. rate is twice as concentrated as SVD Orders rate.
Viroshield	25	Glutaraldehyde plus QAC	Comments as for Omnicide. SVD Orders rate is 300, G.O. rate is 12× more concentrated than SVD Orders rate.

*Products where information on the active components is readily available *and* there is suitable published data to assess the effect of these on African swine fever virus.

†General Orders; millilitres water per millilitre or gram of disinfectant.

‡Gabbert and Neilan, 2020.

ASFV, African swine fever virus; FMD(V), foot and mouth disease (virus); SVD(V), swine vesicular disease (virus); NDV, Newcastle disease virus; SE, *Salmonella* ser. Enteritidis; G.O., General orders; QAC, quaternary ammonium compound.

‘Aldehyde plus QAC’ Defra G.O.-approved products for which quantitative information on composition is readily available are listed in Table 5. It is worth noting that at G.O. concentrations the glutaraldehyde concentration in all these products is at least double the value (0.1%) that was found to reduce ASFV titre by four log_10_ cycles in a 30 min room temperature suspension test with moderate soil [50]. This includes glutaraldehyde-containing products without an SVD Orders approval, which are consequently not included in Table 4.

**Table 5.** Glutaraldehyde content of certain Defra approved disinfectants at General Orders concentrations

Product	Other biocidal component(s)	G.O. dilution rate*	In-use glutaraldehyde concn. (%)
Bioshield P	QAC	40	0.34 %
GPC8	QAC, surfactant	44	0.28 %
InterCID	Formaldehyde	40	0.24 %
Superkill Max	QAC, formaldehyde	10	0.45 %
Viroguard	QAC, formaldehyde	10	0.45 %
Viroshield	QAC	25	0.58 %
Omnicide	QAC	50	0.30 %
Omnicide FG	QAC	50	≥0.20 %
Omnicide FGII	QAC	50	≥0.20 %

*General Orders; millilitres water per millilitre or gram of disinfectant.

### Data gaps

Amongst disinfectants with readily-identifiable chemistries, those using peracetic acid (PAA), constitute the largest group where there are insufficient data to estimate confidently the efficacy against ASFV at G.O. concentrations. The effect of PAA on parvoviruses provides some pointers to the potential efficacy of this agent against ASFV at G.O. concentrations, but the evidence is indirect and requires an assumption that ASFV is no more resistant to PAA than the less hardy varieties of parvovirus. This seems a reasonable assumption, given that parvoviruses have the physical characteristics (small, non-enveloped) that are associated with low susceptibility to surface disinfectants [80]. However, PAA appears to be more active against parvoviruses than many other disinfectants [81], so proper comparative data is needed for clarity.

Some SVD Orders-approved glutaraldehyde products can cautiously be expected to show efficacy against ASFV. However, the key data needed to bolster confidence in recommending glutaraldehyde products (typically glutaraldehyde plus QAC) more broadly at G.O. concentrations is a comparison between susceptibilities to this agent of ASFV and *S.* Enteritidis. The confidence with which products based principally on QAC can be recommended is seriously limited by the lack of data comparing the effect of the same QAC compound upon ASFV and other targets, particularly *S.* Enteritidis and FMDV. This is partly a consequence of the variety of QAC in common use.

Peroxygen-based products (including Virkon S, for which there is a uniquely large amount of experimental data for a commercial product) can cautiously be expected to be effective against ASFV at G.O. concentration (1%), although, as for glutaraldehyde, a comparison with *S.* Enteritidis is lacking. The findings of Gabbert and Neilan [70] suggest potential issues with efficacy of this product on concrete surfaces after shorter contact times. Finally, many products based on phenols and/or cresols cannot currently be assessed for ASFV efficacy at G.O. concentrations because of a lack of experimental data.

## Conclusions

ASFV, having spread out of its historical endemic territory, is now a major challenge to pig production in Europe and Asia. This results from the severity of ASF disease, coupled with the present lack of an effective vaccine, the existence of an infection reservoir in wild and free-ranging suid species, and the ability of the virus to persist at infectious doses in faeces and body fluids, on surfaces and in matrices such as soil. To prevent and control the spread of ASFV, it is critical to have confidence in disinfection to achieve routine biosecurity and to decontaminate premises and vehicles. The UK’s Defra disinfectant approval tests for Disease Control Orders provide a context, of well-established tests associated with effectiveness in field outbreaks of disease, against which the likely efficacy of certain disinfectant products against ASFV may be assessed. Suitable data for some such comparisons have been found in the literature, and on this basis there is fair evidence for the likely efficacy of disinfectants based on sodium hypochlorite and glutaraldehyde, plus a smaller set of data suggesting efficacy of potassium peroxymonosulphate- and iodine-based products. In this context, glutaraldehyde based disinfectants are likely to be a suitable choice for routine and reactive use to control ASF at their Defra General Orders concentrations. Such products have the advantages of lack of corrosiveness and minimal inactivation by organic matter that may otherwise compromise the use of halide, QAC and acidic biocides. However, there remain many gaps that limit predictions for certain major disinfectant classes.

## Supplementary Data

Supplementary material 1
