# Supplementary material for: Disinfection to control African swine fever virus: a UK perspective
Source: J Med Microbiol. 2021 Sep 21;70(9):001410. doi: 10.1099/jmm.0.001410 (PMC8697514; doi:10.1099/jmm.0.001410)
Supplement: Supplementary material 1 [file jmm-70-1410-s001.pdf]

Supplementary Material

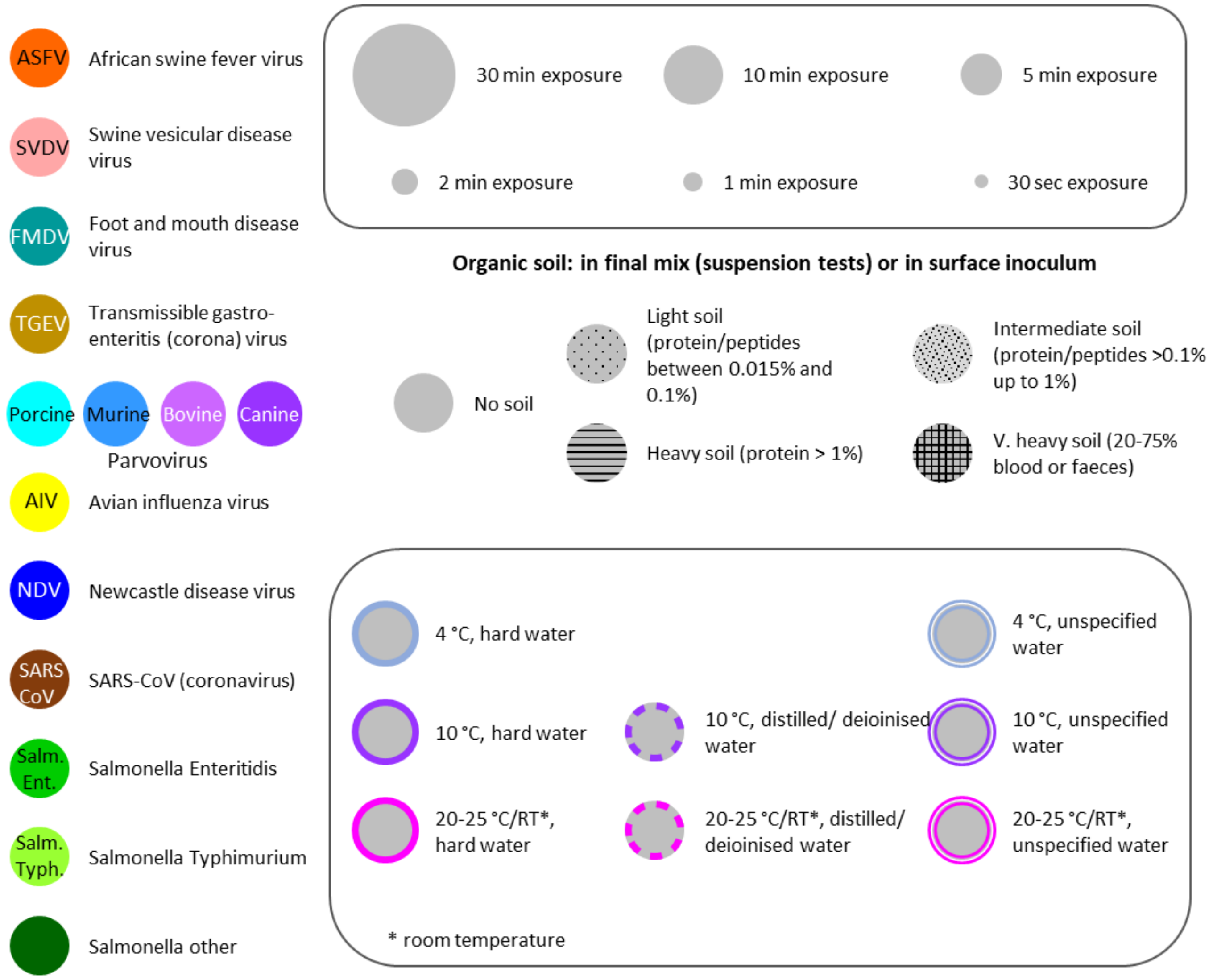

Figure A: Key to visual encoding in all other figures, of target identity, exposure time, organic soil, temperature and diluting/suspending water quality

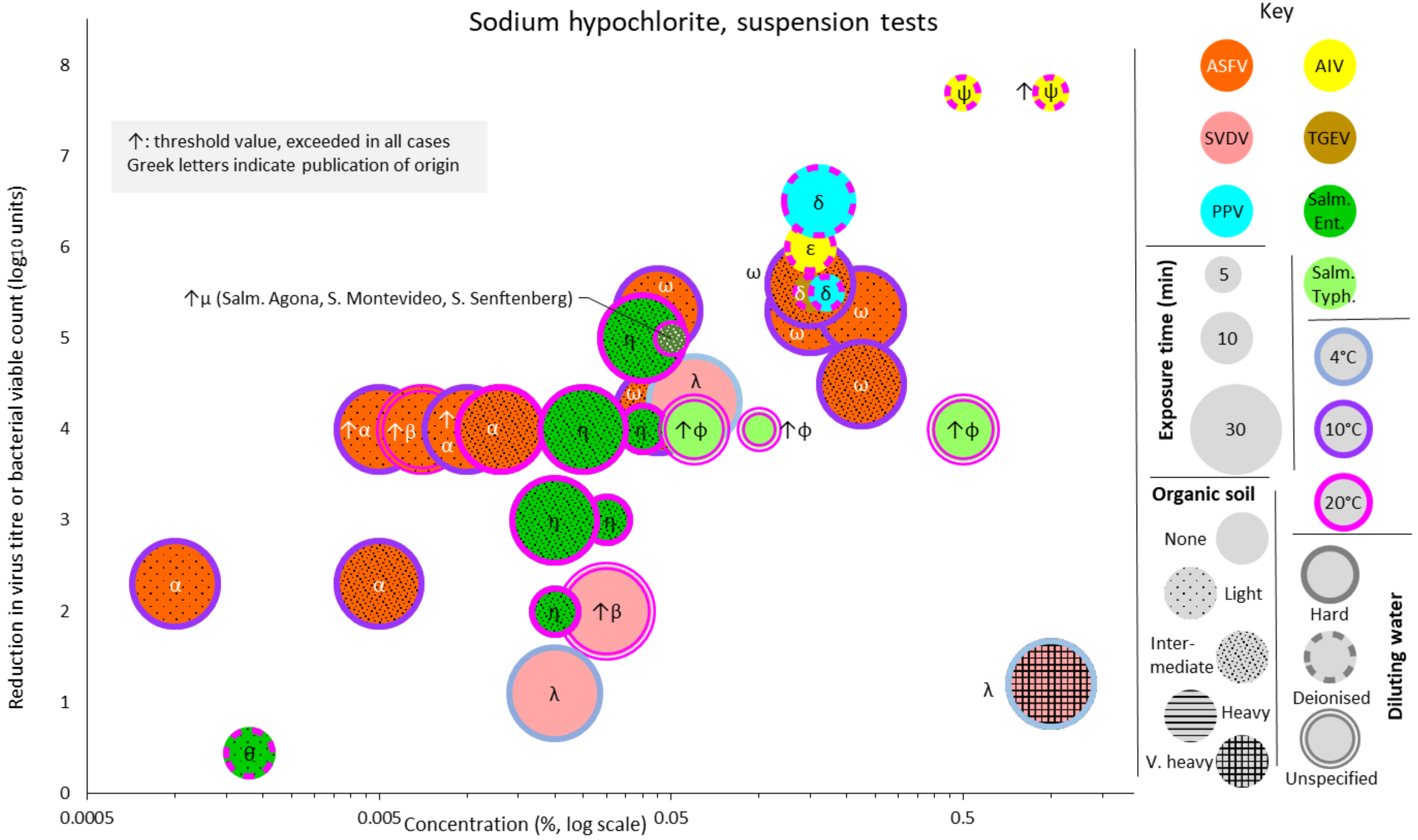

α: Juszkiewicz et al., 2019. β: Shirai et al., 2000. δ: Brown, 1981. ε: Wanaratana et al., 2010. η: Kusumaningrum et al., 2003. θ: Koivunen & Heinonen-Tanski, 2005. λ: Herniman et al., 1973. μ: Møretrø et al., 2009. ϕ: Kich et al., 2004. ψ: Zou et al., 2013. ω: Juszkiewicz et al., 2020

Figure B

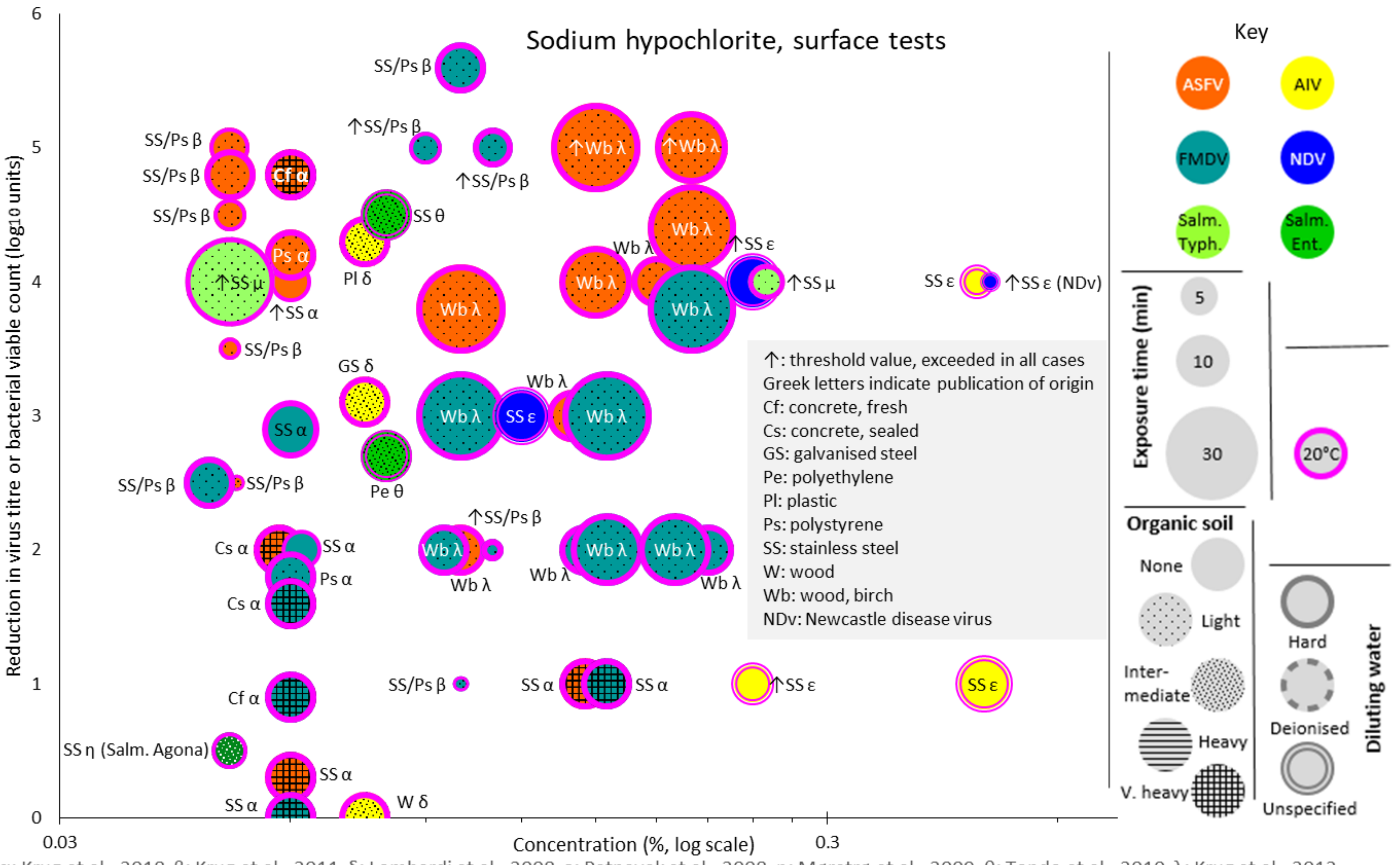

α: Krug et al., 2018. β: Krug et al., 2011. δ: Lombardi et al., 2008. ε: Patnayak et al., 2008. η: Møretrø et al., 2009. θ: Tondo et al., 2010. λ: Krug et al., 2012. µ: Geber et al.2019.

Figure C

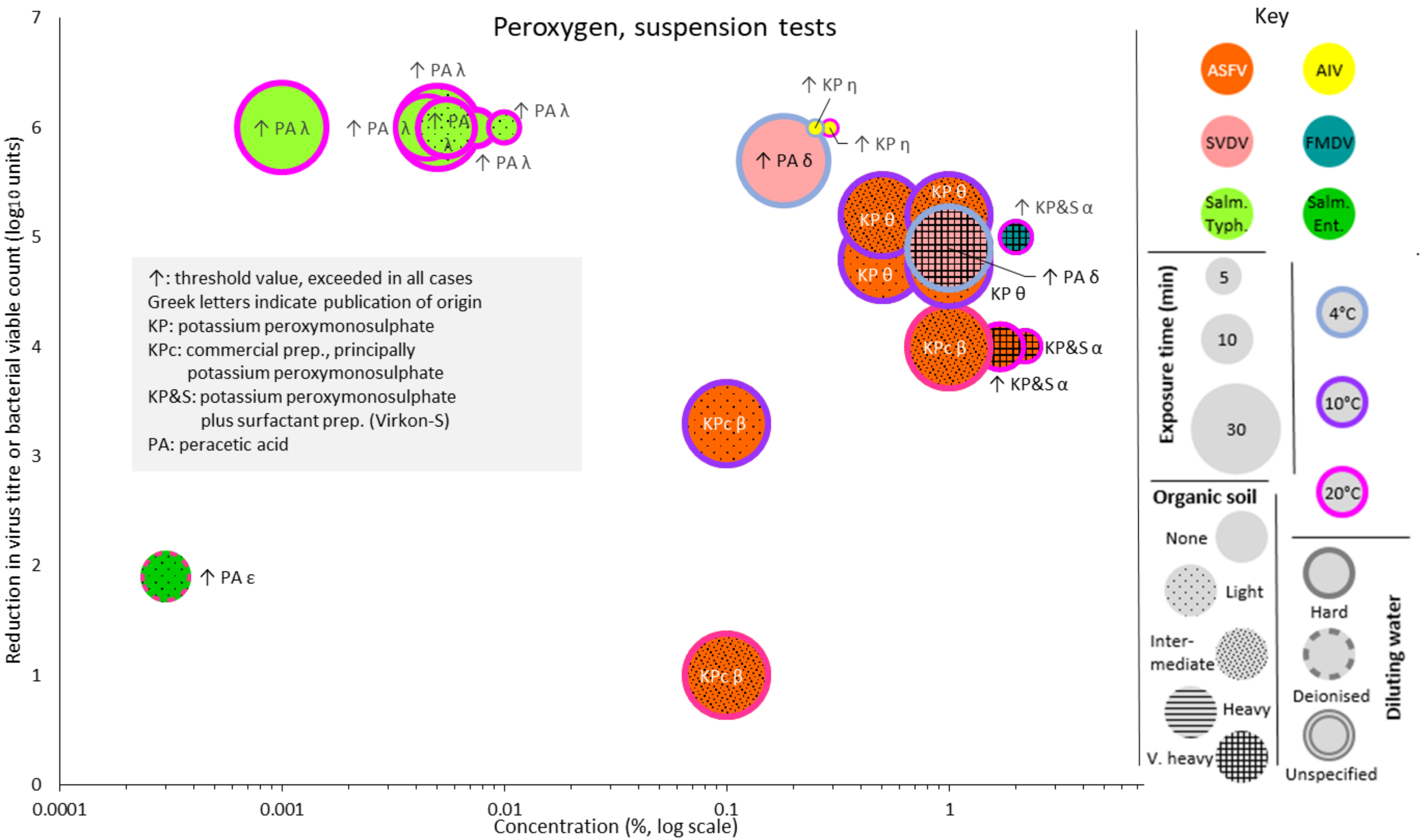

α: Krug et al., 2018. β: Juszkiewicz et al., 2019. δ: Herniman et al., 1973. ε: Koivunen & Heinonen-Tanski, 2005. η: Jang et al., 2014. θ: Juszkiewicz et al., 2020, λ: Geber, 2019.

Figure D

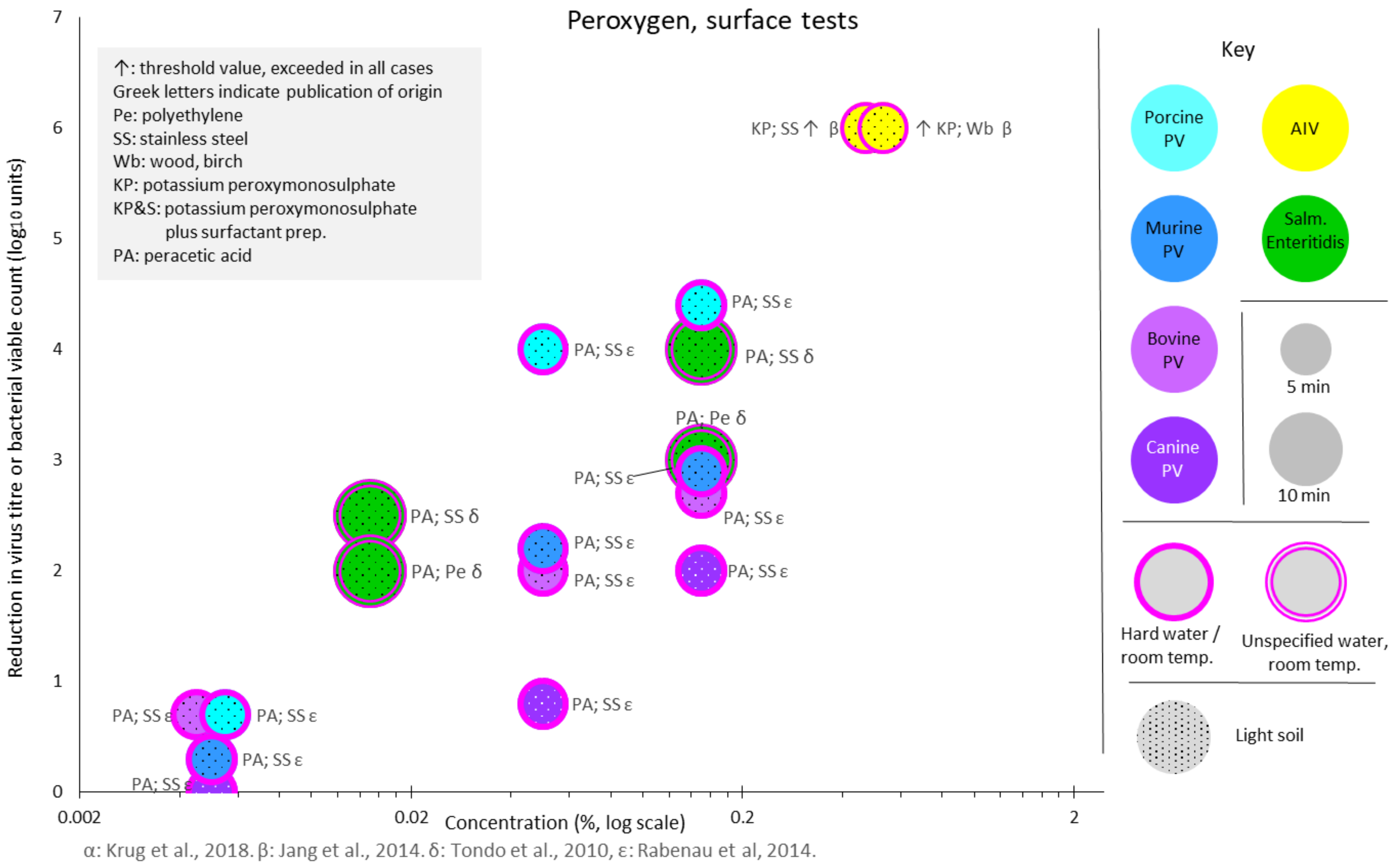
Peroxygen, surface tests
↑: threshold value, exceeded in all cases
Greek letters indicate publication of origin
Pe: polyethylene
SS: stainless steel
Wb: wood, birch
KP: potassium peroxymonosulphate
KP&S: potassium peroxymonosulphate plus surfactant prep.
PA: peracetic acid
KP; SS ↑ β
↑ KP; Wb β
PA; SS ε
PA; SS ε
PA; SS δ
PA; Pe δ
PA; SS ε
PA; SS ε
PA; SS δ
PA; Pe δ
PA; SS ε
PA; SS ε
PA; SS ε
PA; SS ε
PA; SS ε
PA; SS ε
PA; SS ε
PA; SS ε
Reduction in virus titre or bacterial viable count (log10 units)
Concentration (%, log scale)
0
1
2
3
4
5
6
7
0.002
0.02
0.2
2
Key
Porcine PV
AIV
Murine PV
Salm. Enteritidis
Bovine PV
Canine PV
5 min
10 min
Hard water / room temp.
Unspecified water, room temp.
Light soil
α: Krug et al., 2018. β: Jang et al., 2014. δ: Tondo et al., 2010, ε: Rabenau et al, 2014.

Figure E

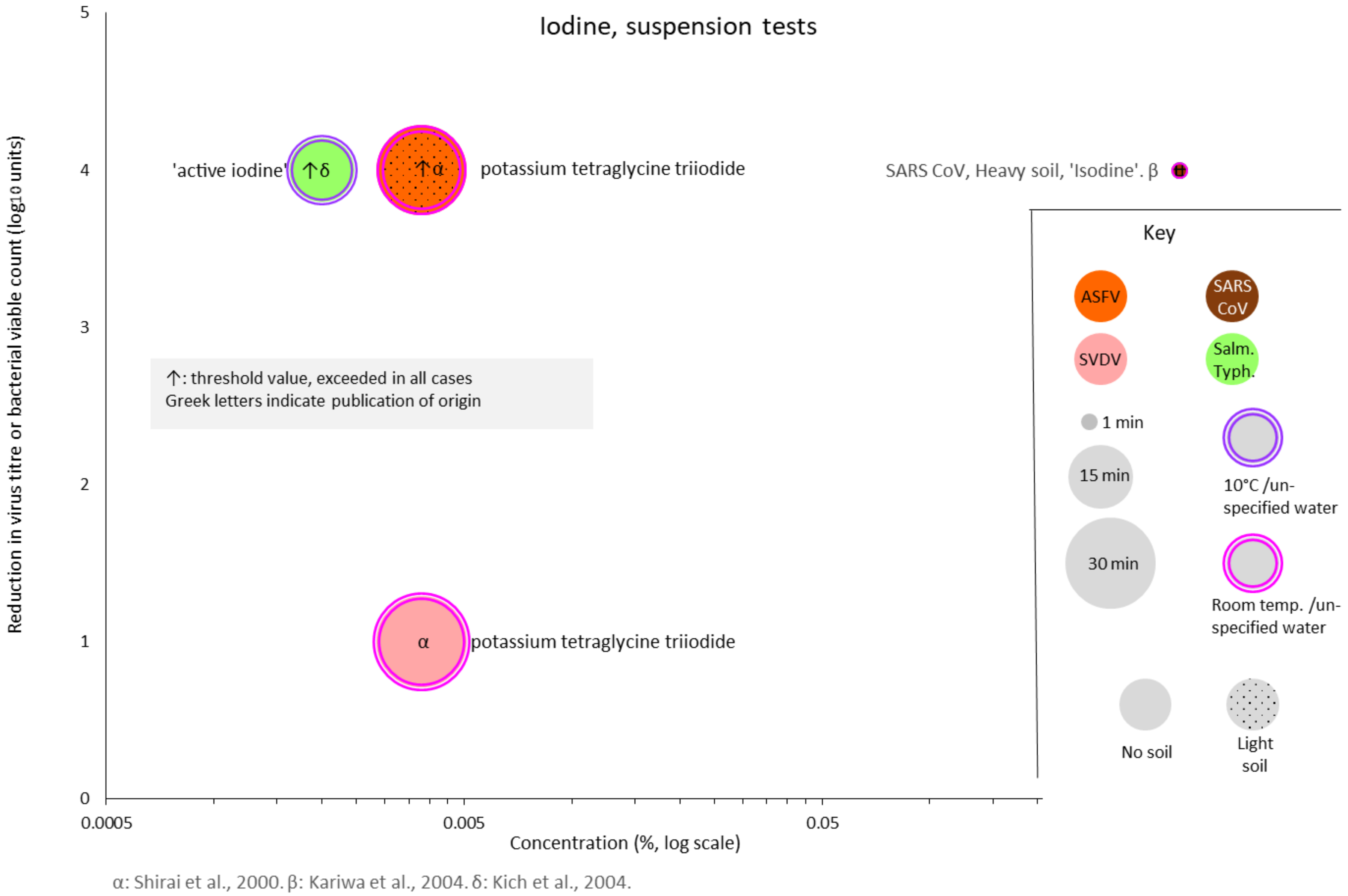
Iodine, suspension tests
Reduction in virus titre or bacterial viable count (log10 units)
5
4
3
2
1
0
'active iodine'
↑δ
↑α
potassium tetraglycine triiodide
SARS CoV, Heavy soil, 'Isodine'. β
↑: threshold value, exceeded in all cases
Greek letters indicate publication of origin
α
potassium tetraglycine triiodide
Key
ASFV
SARS CoV
SVDV
Salm. Typh.
1 min
15 min
30 min
10°C /un-specified water
Room temp. /un-specified water
No soil
Light soil
0.0005
0.005
0.05
Concentration (%, log scale)
α: Shirai et al., 2000. β: Kariwa et al., 2004. δ: Kich et al., 2004.

Figure F

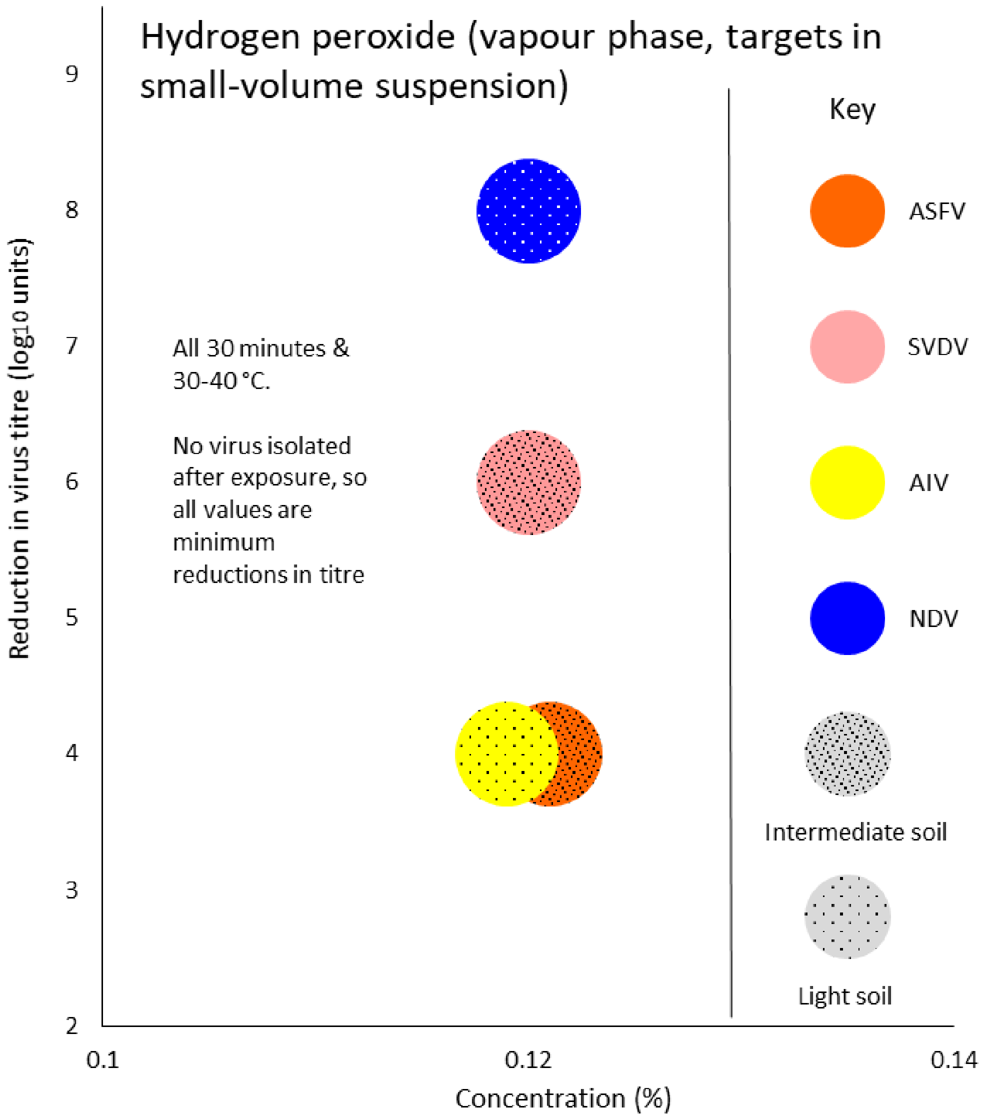

Source: Heckert et al., 1997

Figure G

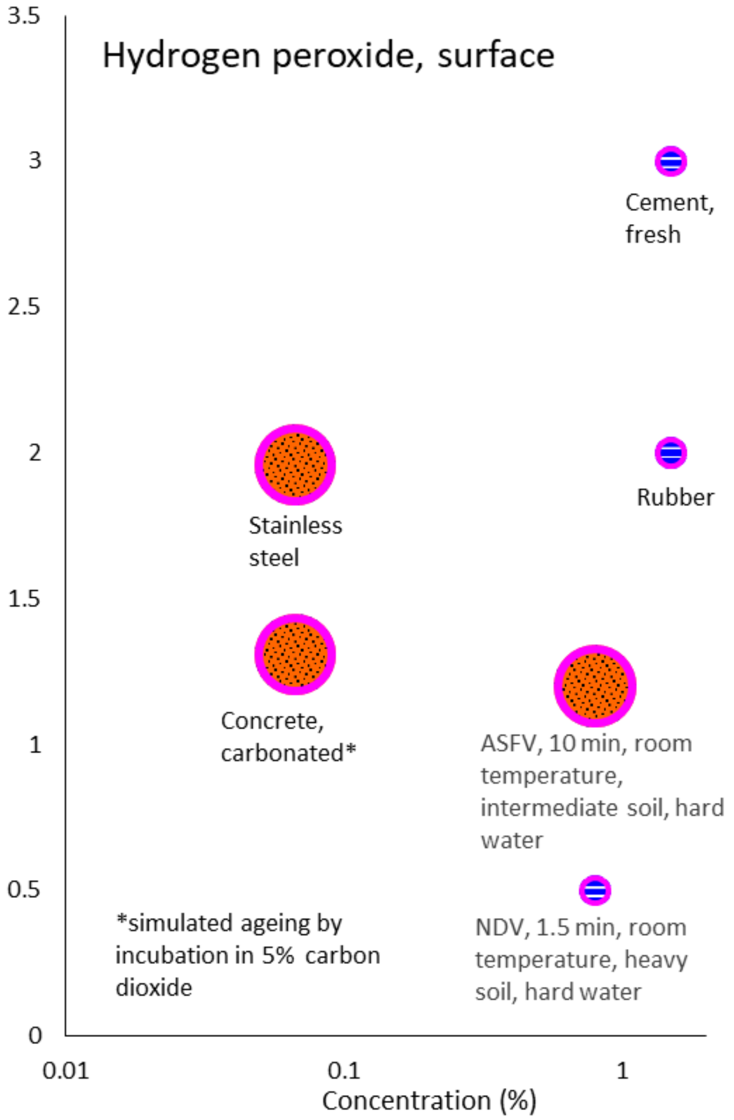

Sources: Gabbert and Neilan, 2020 (ASFV) & Gamal, 2018 (NDV).

Figure H

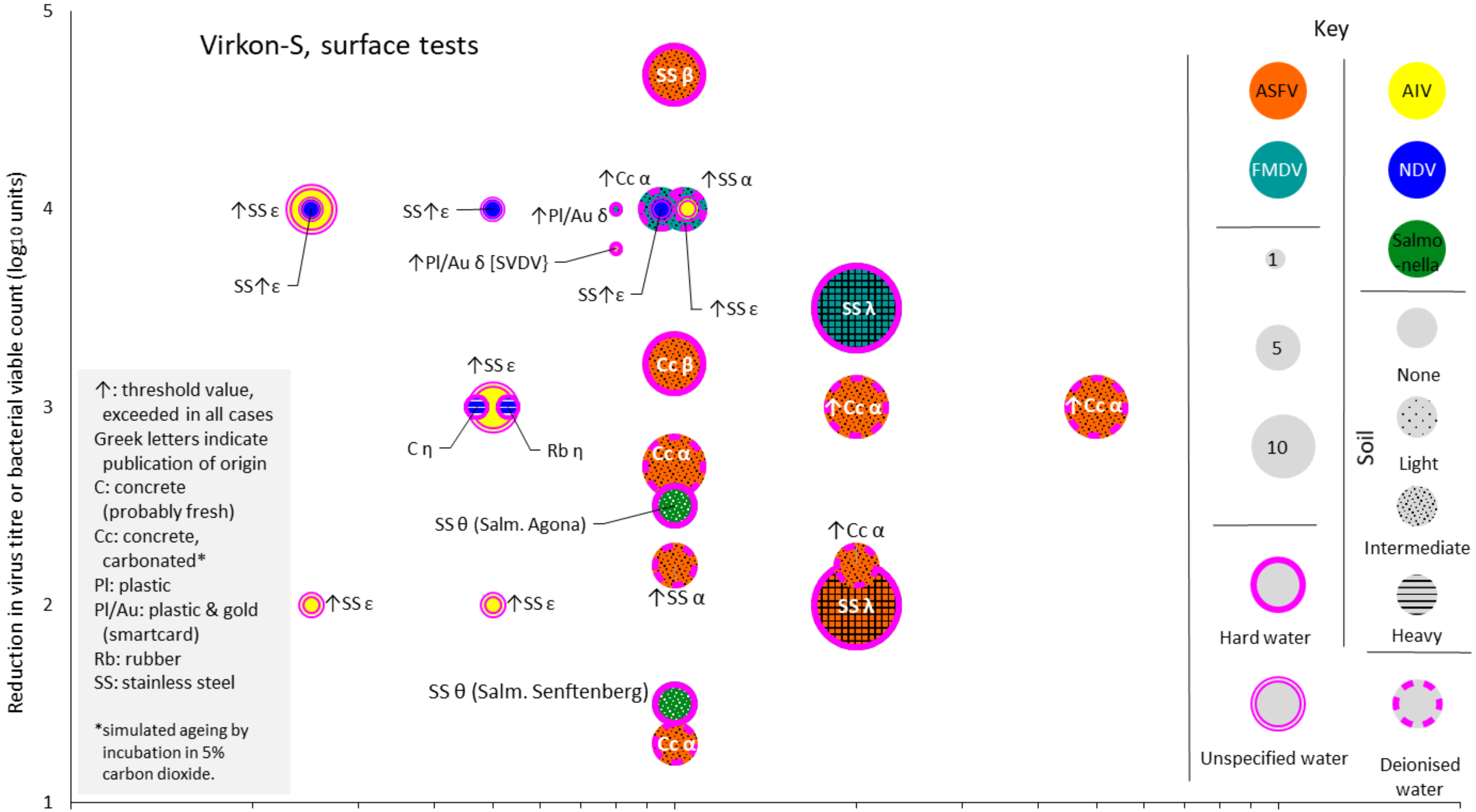

α: Gabbert et al., 2020. β: Gabbert and Neilan, 2020. δ: Gabbert et al., 2018. ε: Patnayak et al., 2008. η: Gamal et al., 2018. θ: Møretrø et al., 2009. λ: Krug et al., 2018.

Figure I

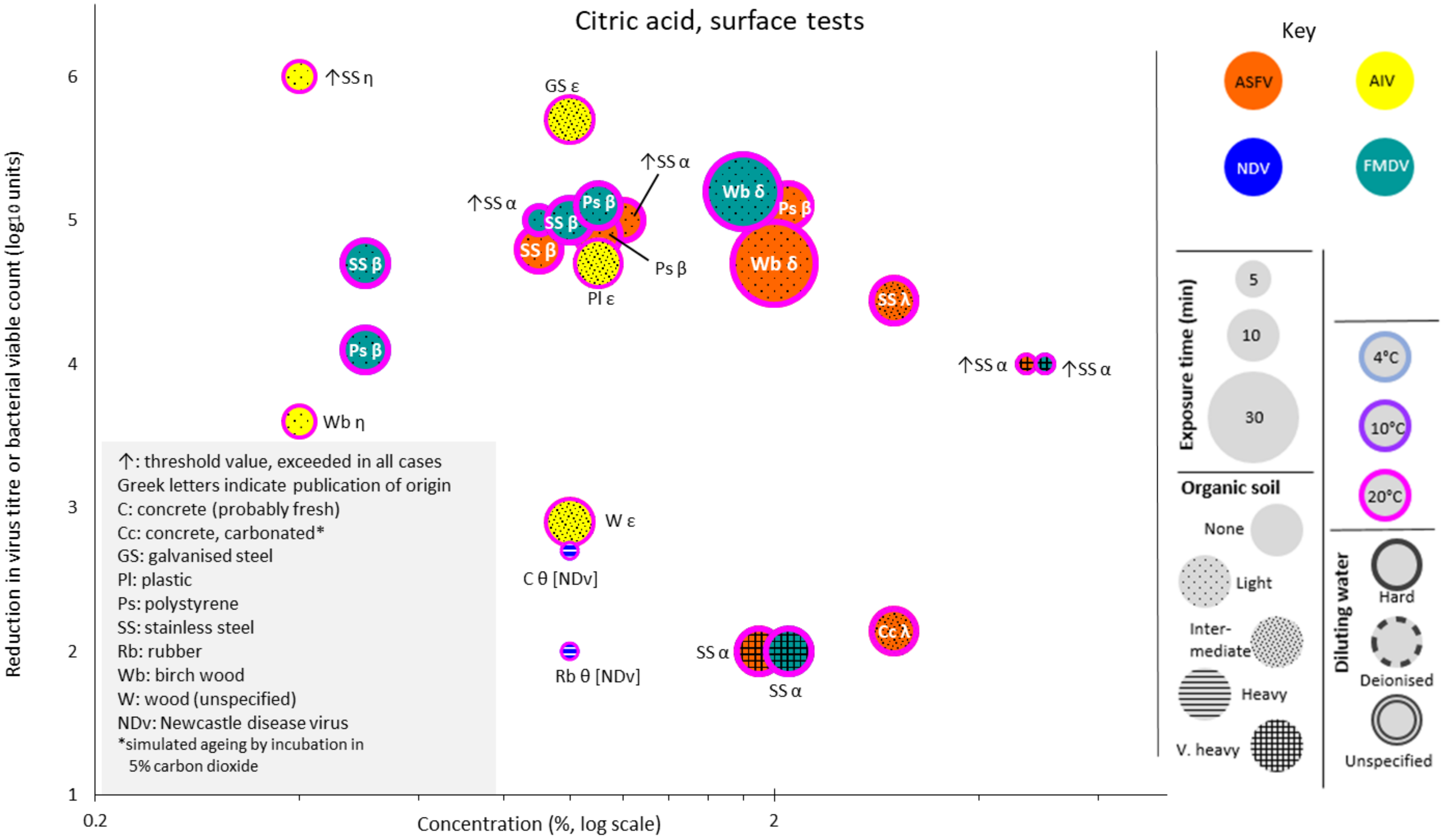

α: Krug et al., 2018. β: Krug et al., 2011. δ: Krug et al., 2012. ε: Lombardi et al., 2008. η: Jang et al., 2014. θ: Gamal et al., 2018. λ: Gabbert and Neilan, 2020.

Figure J

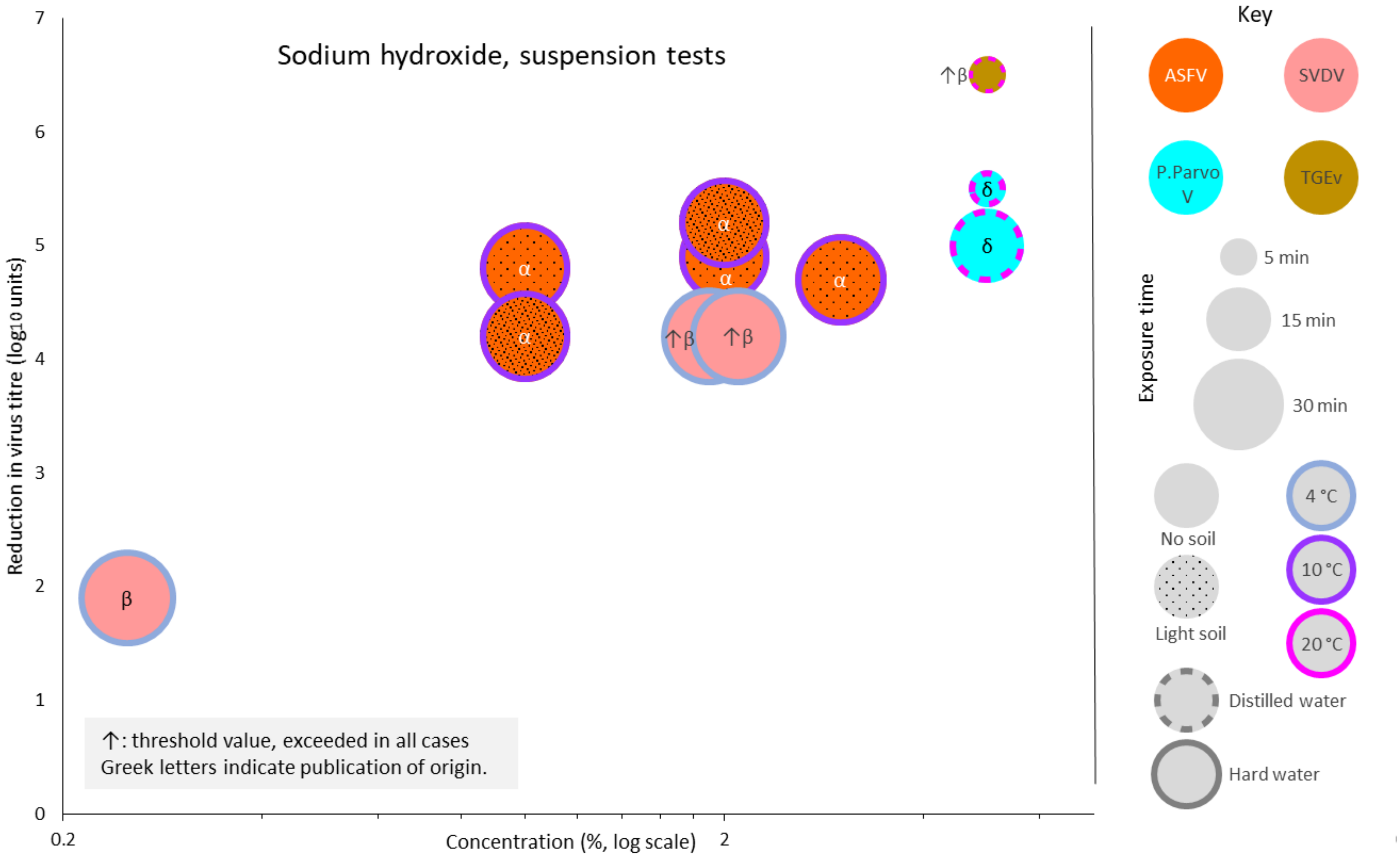

α: Juszkiewicz et al., 2020. β: Herniman et al., 1973. δ: Brown, 1981.

Figure K

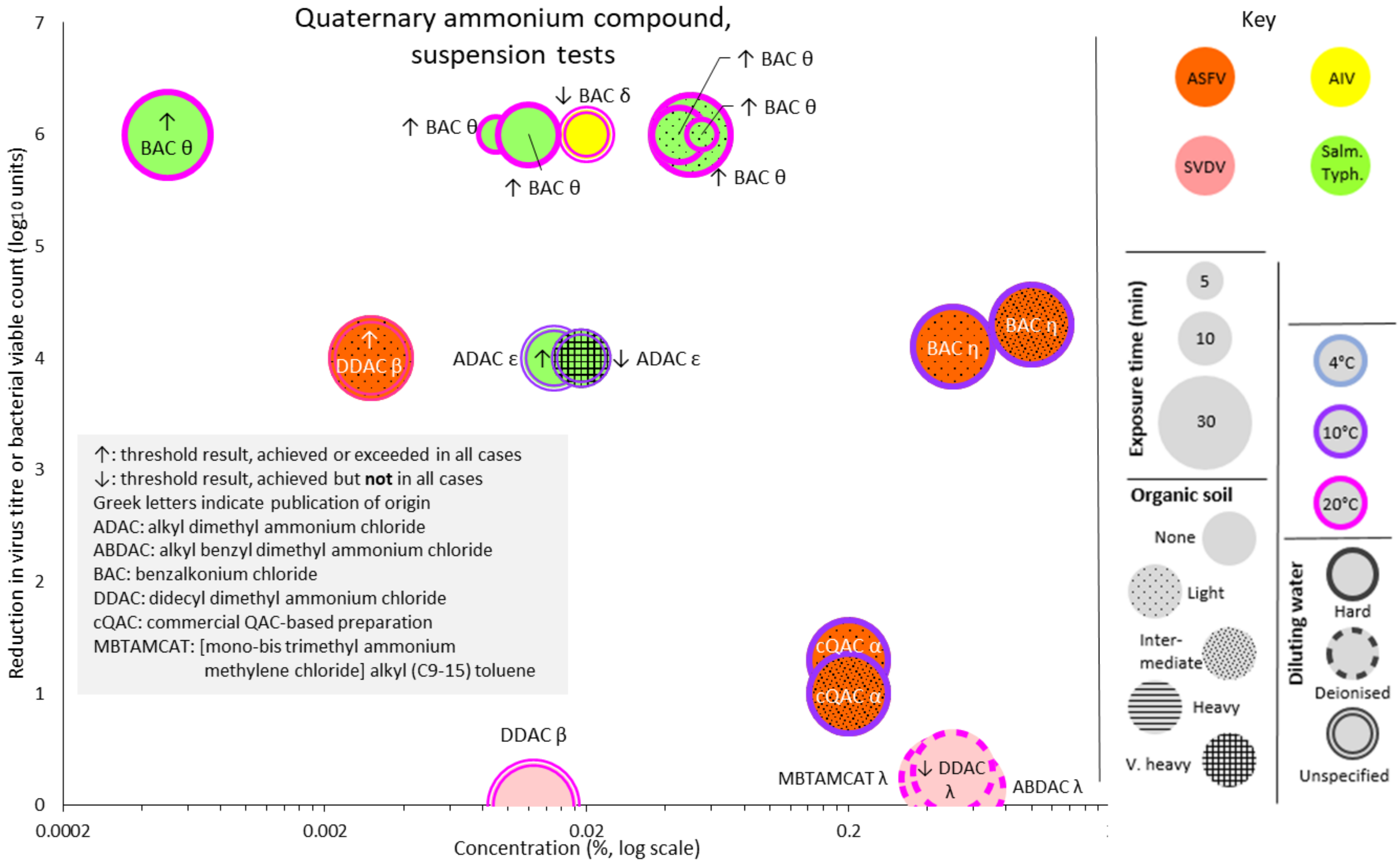

α: Juszkiewicz et al., 2019. β: Shirai et al., 2000. δ: Wanaratana et al., 2010. ε: Kich et al., 2004. η: Juszkiewicz et al., 2020, θ: Geber 2019. λ: Shirai et al, 1997.

Figure L

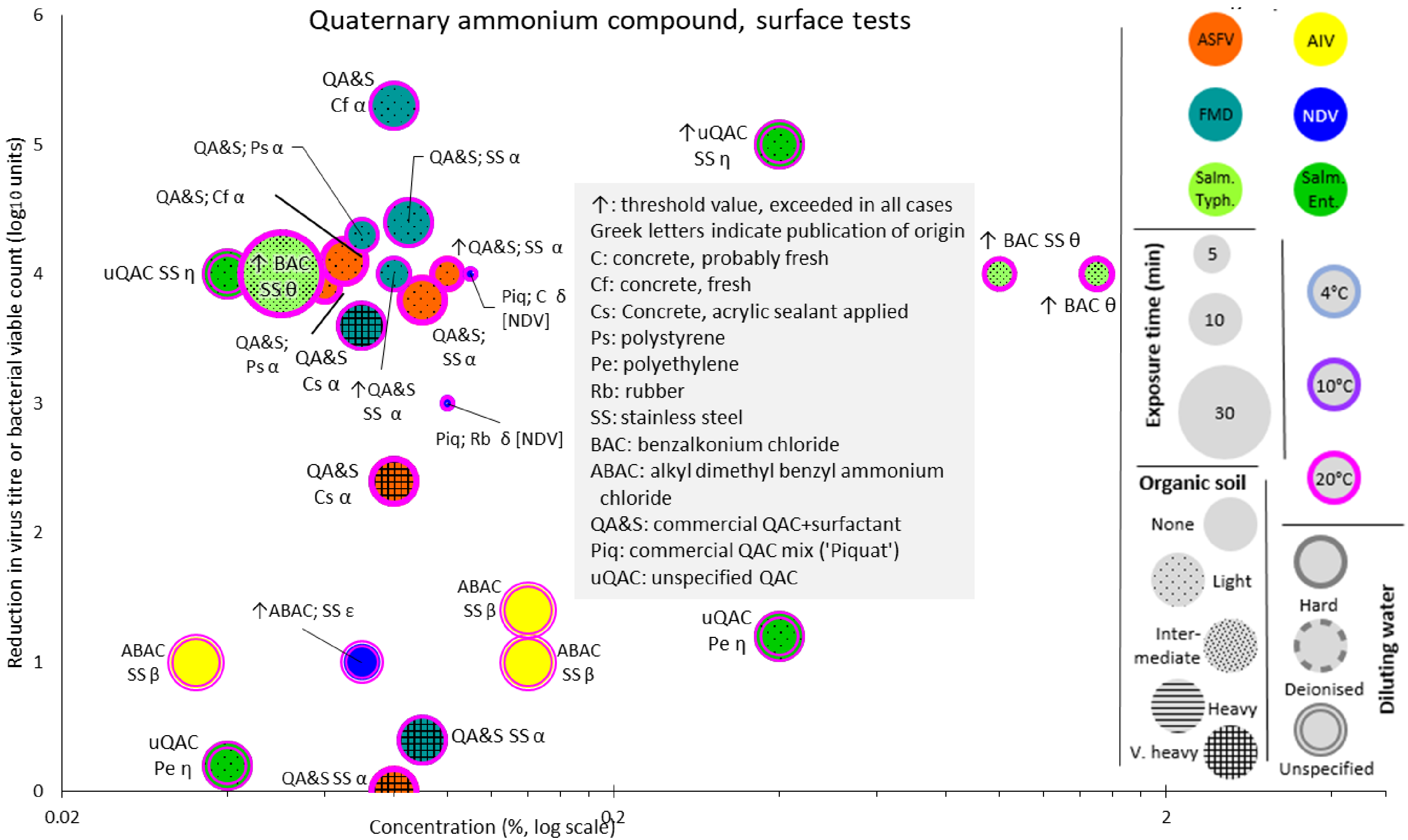

α: Krug et al., 2018. β: Shirai et al., 2000. δ: Wanaratana et al., 2010. ε: Patnayak et al., 2008. η: Tondo et al., 2010, θ: Geber 2019.

Figure M

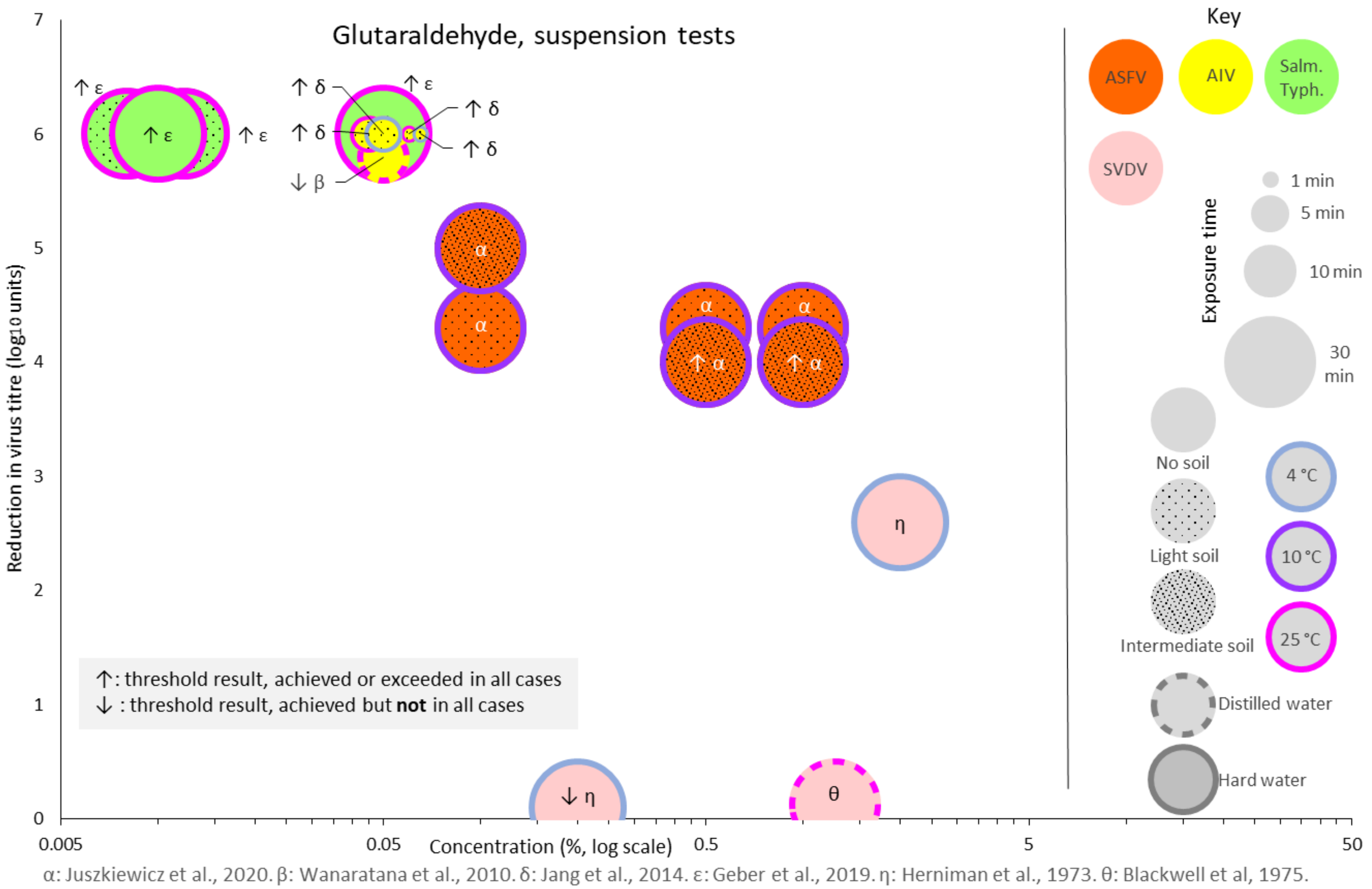
Glutaraldehyde, suspension tests
Reduction in virus titre (log10 units)
Concentration (%, log scale)
0.005
0.05
0.5
5
50
0
1
2
3
4
5
6
7
↑ ε
↑ δ
↓ β
α
↑ α
η
↓ η
θ
↑: threshold result, achieved or exceeded in all cases
↓ : threshold result, achieved but **not** in all cases
Key
ASFV
AIV
Salm. Typh.
SVDV
Exposure time
1 min
5 min
10 min
30 min
No soil
Light soil
Intermediate soil
Distilled water
Hard water
4 °C
10 °C
25 °C
α: Juszkiewicz et al., 2020. β: Wanaratana et al., 2010. δ: Jang et al., 2014. ε: Geber et al., 2019. η: Herniman et al., 1973. θ: Blackwell et al, 1975.

Figure N

References cited in figures B to N

Blackwell JH, Graves JH, McKercher PD. Chemical inactivation of swine vesicular disease virus. *British Veterinary Journal* 1975;131:317–323: doi: 10.1016/S0007-1935(17)35288-0

Brown TT. Laboratory evaluation of selected disinfectants as virucidal agents against porcine parvovirus, pseudorabies virus, and transmissible gastroenteritis virus. *Am J Vet Res* 1981;42:1033–1036

Gabbert LR, Smith JD, Neilan JG, Ferman GS, Rasmussen MV. Smart card decontamination in a high-containment laboratory. *Health Security* 2018;16:244–251: doi: 10.1089/hs.2018.0023

Gabbert LR, Neilan J. Disinfectants against ASF virus: efficacy evaluation. Online Conference: *African swine fever: An unprecedented global threat - A challenge to livelihoods, food security and biodiversity. Call for action.* WHO & OIE. http://www.gf-tads.org/events/events-detail/en/c/1152886/ (accessed 16 June 2021)

Gabbert LR, Neilan JG, Rasmussen M. Recovery and chemical disinfection of foot-and-mouth disease and African swine fever viruses from porous concrete surfaces. *Journal of Applied Microbiology* 2020;129:1092–1101: doi: 10.1111/jam.14694

Gamal AM, Rohaim MA, Helal AM, Hamoud MM, Zaki MM, *et al.* Evaluation of the viricidal efficacy of commercially used disinfectants against Newcastle Disease virus. *Biosci Res* 2018;15:3283–3292

Geber F, Reinhardt M, Kreuz M, Cuny C, Pfeifer Y, *et al.* A comparison of different methods to determine disinfectant susceptibility of multidrug-resistant bacteria. *Berliner und Münchener Tierärztliche Wochenschrift* 2019;132:367–376: doi: 10.2376/0005-9366-18047

Heckert RA, Best M, Jordan LT, Dulac GC, Eddington DL, *et al.* Efficacy of vaporized hydrogen peroxide against exotic animal viruses. *Appl Environ Microbiol* 1997;63:3916–3918

Herniman KA, Medhurst PM, Wilson JN, Sellers RF. The action of heat, chemicals and disinfectants on swine vesicular disease virus. *Veterinary Record* 1973;93:620–624: doi: 10.1136/vr.93.24.620

Jang Y, Lee J, So B, Lee K, Yun S, *et al.* Evaluation of changes induced by temperature, contact time, and surface in the efficacies of disinfectants against avian influenza virus. *Poult Sci* 2014;93:70–76: doi: 10.3382/ps.2013-03452

Juszkiewicz M, Walczak M, Mazur-Panasiuk N, Wozniakowski G. Virucidal effect of chosen disinfectants against African swine fever virus (ASFV) - preliminary studies. *Pol J Vet Sci* 2019;22:777–780: doi: 10.24425/pjvs.2019.131407

Juszkiewicz M, Walczak M, Mazur-Panasiuk N, Woźniakowski G. Effectiveness of chemical compounds used against African swine fever virus in commercial available disinfectants. *Pathogens* 2020;9:article 878: doi: 10.3390/pathogens9110878

Kariwa H, Fujii N, Takashima I. Inactivation of SARS coronavirus by means of povidone-iodine, physical conditions, and chemical reagents. *Japanese Journal of Veterinary Research* 2004;52:105–112

Kich JD, Borowsky LM, Silva VS, Ramenzoni M, Triches N, *et al.* Avaliação da atividade antibacteriana de seis desinfetantes comerciais frente a amostras de *Salmonella* Typhimurium isoladas de suínos [Evaluation of the antibacterial activity of six commercial disinfectants against *Salmonella* Typhimurium strains isolated from swine]. *Acta Scientiae Veterinariae* 2004;32: 33-39

Koivunen J, Heinonen-Tanski H. Inactivation of enteric microorganisms with chemical disinfectants, UV irradiation and combined chemical/UV treatments. *Water Research* 2005;39:1519–1526: doi: 10.1016/j.watres.2005.01.021

Krug PW, Lee LJ, Eslami AC, Larson CR, Rodriguez L. Chemical disinfection of high-consequence transboundary animal disease viruses on nonporous surfaces. *Biologicals* 2011;39:231–235: doi: 10.1016/j.biologicals.2011.06.016

Krug PW, Larson CR, Eslami AC, Rodriguez LL. Disinfection of foot-and-mouth disease and African swine fever viruses with citric acid and sodium hypochlorite on birch wood carriers. *Veterinary Microbiology* 2012;156:96–101: doi: 10.1016/j.vetmic.2011.10.032

Krug PW, Davis T, O'Brien C, LaRocco M, Rodriguez LL. Disinfection of transboundary animal disease viruses on surfaces used in pork packing plants. *Veterinary Microbiology* 2018;219:219–225: doi: 10.1016/j.vetmic.2018.04.029

Kusumaningrum HD, Paltinaite R, Koomen AJ, Hazeleger WC, Rombouts FM, *et al.* Tolerance of *Salmonella* Enteritidis and *Staphylococcus aureus* to surface cleaning and household bleach. *J Food Prot* 2003;66:2289–2295: doi: 10.4315/0362-028X-66.12.2289

Lombardi ME, Ladman BS, Alphin RL, Benson ER. Inactivation of avian influenza virus using common detergents and chemicals. *Avian Diseases* 2008;52:118–123: doi: 10.1637/8055-070907-Reg

Møretrø T, Vestby LK, Nesse LL, Storheim SE, Kotlarz K, *et al.* Evaluation of efficacy of disinfectants against *Salmonella* from the feed industry. *J Appl Microbiol* 2009;106:1005–1012: doi: 10.1111/j.1365-2672.2008.04067.x

Patnayak DP, Prasad M, Malik YS, Ramakrishnan MA, Goyal SM. Efficacy of disinfectants and hand sanitizers against avian respiratory viruses. *Avian Diseases* 2008;52:199–202: doi: 10.1637/8097-082807-Reg.1

Rabenau HF, Steinmann J, Rapp I, Schwebke I, Eggers M. Evaluation of a virucidal quantitative carrier test for surface disinfectants. *PLoS ONE* 2014;9:e86128: doi: 10.1371/journal.pone.0086128

Shirai J, Kanno T, Inoue T, Mitsubayashi S, Seki R. Effects of quaternary ammonium compounds with 0.1% sodium hydroxide on swine vesicular disease virus. *J Vet Med Sci* 1997;59:323–328: doi: 10.1292/jvms.59.323

Shirai J, Kanno T, Tsuchiya Y, Mitsubayashi S, Seki R. Effects of chlorine, iodine, and quaternary ammonium compound disinfectants on several exotic disease viruses. *Journal of Veterinary Medical Science* 2000;62:85–92: doi: 10.1292/jvms.62.85

Tondo EC, Machado TRM, Malheiros P da S, Padrão DK, Carvalho AL de, *et al.* Adhesion and biocides inactivation of *Salmonella* on stainless steel and polyethylene. *Brazilian Journal of Microbiology* 2010;41:1027–1037: doi: 10.1590/S1517-83822010000400022

Wanaratana S, Tantilertcharoen R, Sasipreeyajan J, Pakpinyo S. The inactivation of avian influenza virus subtype H5N1 isolated from chickens in Thailand by chemical and physical treatments. *Veterinary Microbiology* 2010;140:43–48: doi: 10.1016/j.vetmic.2009.07.008

Zou S, Guo J, Gao R, Dong L, Zhou J, *et al.* Inactivation of the novel avian influenza A (H7N9) virus under physical conditions or chemical agents treatment. *Virol J* 2013;10:289: doi: 10.1186/1743-422X-10-289
